# SLPI Inhibits ATP-Mediated Maturation of IL-1β in Human Monocytic Leukocytes: A Novel Function of an Old Player

**DOI:** 10.3389/fimmu.2019.00664

**Published:** 2019-04-04

**Authors:** Anna Zakrzewicz, Katrin Richter, Dariusz Zakrzewicz, Kathrin Siebers, Jelena Damm, Alisa Agné, Andreas Hecker, J. Michael McIntosh, Walee Chamulitrat, Gabriela Krasteva-Christ, Ivan Manzini, Ritva Tikkanen, Winfried Padberg, Sabina Janciauskiene, Veronika Grau

**Affiliations:** ^1^Laboratory of Experimental Surgery, Department of General and Thoracic Surgery, German Center for Lung Research, Justus-Liebig-University Giessen, Giessen, Germany; ^2^German Center for Lung Research, Faculty of Medicine, Institute of Biochemistry, Justus-Liebig-University Giessen, Giessen, Germany; ^3^Department of Biology, University of Utah, Salt Lake City, UT, United States; ^4^George E. Wahlen Veterans Affairs, Medical Center, Salt Lake City, UT, United States; ^5^Department of Psychiatry, University of Utah, Salt Lake City, UT, United States; ^6^Department of Internal Medicine IV, University Heidelberg Hospital, Heidelberg, Germany; ^7^Faculty of Medicine, Institute of Anatomy and Cell Biology, Saarland University, Homburg, Germany; ^8^Department of Animal Physiology and Molecular Biomedicine, Justus-Liebig-University Giessen, Giessen, Germany; ^9^Faculty of Medicine, Institute of Biochemistry, Justus-Liebig-University, Giessen, Germany; ^10^Department of Respiratory Medicine, German Center for Lung Research, Hannover Medical School, Hannover, Germany

**Keywords:** annexin II, calcium-independent phospholipase A2β, caspase-1, IL-1β, inflammasome, SLPI, nAChR, P2X_7_ receptor

## Abstract

Interleukin-1β (IL-1β) is a potent, pro-inflammatory cytokine of the innate immune system that plays an essential role in host defense against infection. However, elevated circulating levels of IL-1β can cause life-threatening systemic inflammation. Hence, mechanisms controlling IL-1β maturation and release are of outstanding clinical interest. Secretory leukocyte protease inhibitor (SLPI), in addition to its well-described anti-protease function, controls the expression of several pro-inflammatory cytokines on the transcriptional level. In the present study, we tested the potential involvement of SLPI in the control of ATP-induced, inflammasome-dependent IL-1β maturation and release. We demonstrated that SLPI dose-dependently inhibits the ATP-mediated inflammasome activation and IL-1β release in human monocytic cells, without affecting the induction of pro-IL-1β mRNA by LPS. In contrast, the ATP-independent IL-1β release induced by the pore forming bacterial toxin nigericin is not impaired, and SLPI does not directly modulate the ion channel function of the human P2X_7_ receptor heterologously expressed in *Xenopus laevis* oocytes. In human monocytic U937 cells, however, SLPI efficiently inhibits ATP-induced ion-currents. Using specific inhibitors and siRNA, we demonstrate that SLPI activates the calcium-independent phospholipase A2β (iPLA2β) and leads to the release of a low molecular mass factor that mediates the inhibition of IL-1β release. Signaling involves nicotinic acetylcholine receptor subunits α7, α9, α10, and Src kinase activation and results in an inhibition of ATP-induced caspase-1 activation. In conclusion, we propose a novel anti-inflammatory mechanism induced by SLPI, which inhibits the ATP-dependent maturation and secretion of IL-1β. This novel signaling pathway might lead to development of therapies that are urgently needed for the prevention and treatment of systemic inflammation.

## Introduction

Interleukin-1β (IL-1β) is a potent, multifunctional, pro-inflammatory cytokine of the innate immune system that is essential for host-response and resistance to pathogens (1). Nevertheless, excessive systemic levels of IL-1β are involved in the pathogenesis of various inflammatory disorders, including rheumatoid arthritis, gout, transplant rejection, periodic fever, systemic inflammatory response syndrome (SIRS), sepsis, and multi organ dysfunction syndrome (MODS) (2–4). To avoid severe adverse effects, the release of IL-1β is firmly controlled and most often requires two consecutive danger signals. Toll-like receptor agonists such as lipopolysaccharide (LPS) are prototypical first signals (signal 1) initiating the synthesis of the inactive IL-1β precursor (pro-IL-1β) via nuclear factor-κB (NF-κB)-dependent signal transduction (5). Extracellular ATP, mainly released from damaged host cells can act as a second signal (signal 2). ATP activates the purinergic P2X_7_ receptor (P2X_7_R), triggers the efflux of potassium ions out of the cells, the assembly of the cytoplasmic NLRP3 (NACHT, LRR, and PYD domains-containing protein 3) inflammasome, followed by the conversion of pro-caspase-1 into active caspase-1 that is required for the proteolytic cleavage of pro-IL-1β and release of mature IL-1β (6, 7). Dysregulation of any of these steps might account for increased secretion of IL-1β and progression of IL-1β-mediated diseases. Mechanisms controlling IL-1β maturation in the presence of both danger signals are of substantial clinical interest (8).

Purinergic signaling via P2 receptors can lead to both, activation and inhibition of innate, and adaptive immune reactions (9–12). Apart from the above-mentioned activation of NLRP3 inflammasomes, stimulation of P2X_7_R by extracellular ATP, and some other non-purine signaling molecules activates diverse cellular events, such as the autocrine release of cytoplasmic ATP, changes in cellular energy metabolism, release of microparticles, exosomes, and secretory lysosomes, induction of cell death, and stimulation of T cell survival and differentiation (11, 12). Hence, an inhibition of P2X_7_R activation is of high biomedical relevance for the control of IL-1β release and beyond.

Recently, our group proposed a novel cholinergic mechanism that inhibits the ATP-induced inflammasome activation in monocytes (13–15). We demonstrated that canonical agonists of the nicotinic acetylcholine (ACh) receptors (nAChRs) namely ACh, choline (Cho) and nicotine and the common metabolites of phosphatidylcholines including lysophosphatidylcholine, glycerophospocholine, phosphocholine (PC) as well as PC-modified macromolecules efficiently inhibit the ATP-induced release of IL-1β (13–15). The acute phase reactant C-reactive protein potentiates the inhibitory efficiency of PC (16). This inhibitory effect of nAChR agonists is mediated via nAChR subunits α7, α9, and /or α10 (13–15). Interestingly, nAChRs do not function as ligand-gated ion channels in monocytic cells but rather as metabotropic receptors that eventually inhibit the ionchannel function of P2X_7_R (13, 15, 16).

Secretory leukocyte protease inhibitor (SLPI) is a non-glycosylated, basic 11.7 kDa monomeric protein composed of two cysteine rich whey-acidic-protein (WAP) domains. SLPI is constitutively produced and secreted by the majority of mucosal epithelial cells and also by monocytes/macrophages and neutrophils (17). Expression of SLPI can be induced by LPS, IL-1β, IL-6, or tumor necrosis factor-α (TNF-α) and suppressed by interferon-γ (IFN-γ) (18–20). Its physiological concentration differs depending on the fluid analyzed. Systemic levels of SLPI are low, only about 40 ng/ml can be measured in the blood of healthy volunteers, whereas the concentration in saliva or in the epithelial lining fluid of the lung are in the range of 10 μg/ml (21, 22). SLPI possesses a high affinity for serine proteases such as neutrophil elastase, cathepsin G, and trypsin (23). The anti-protease activity of SLPI depends on its active site, which is located at Leu72-Met73 of the C-terminal WAP domain. Any modification of Met73 such as oxidation has been shown to reduce the anti-proteolytic potential of SLPI (24). In addition, SLPI possesses potent anti-microbial and anti-inflammatory properties, which are independent of its primarily described anti-protease function (23, 25–27). The biochemical mechanism responsible for the anti-microbial activities is still unclear. One possible explanation could be the cationic nature of SLPI, which could destabilize the anionic cell membrane of bacteria (23).

The anti-inflammatory properties of SLPI known so far, are related to the inhibition of NF-κB signaling, which can be achieved in several ways. First, SLPI can interfere with the interaction between LPS and CD14, reducing the subsequent release of inflammatory mediators (28). Furthermore, exogenous SLPI can be rapidly internalized by monocytes, where it localizes within the cytoplasm and in the nucleus (29). SLPI can interact with the cytosolic inhibitor of κB (I-κB), prohibit its degradation and consequently prevent NF-κB translocation to the nucleus (30). SLPI can also compete with NF-κB (p65) for binding to the DNA consensus binding site in the promotor region of NF-κB-regulated genes (23–25, 29, 30). Furthermore, stimulation with SLPI was shown to attenuate p38 MAPK activation and increase Akt phosphorylation in rat adult ventricular myocytes (31). SLPI-deficient mice exhibit normal fetal development, survive into adulthood and show no obvious pathological phenotype (32). Interestingly, SLPI-deficient mice challenged with LPS show a lower survival rate compared to wild-type (WT) animals and elevated plasma levels of IL-6, a cytokine that is induced by IL-1β (33). SLPI can negatively regulate the expression of some of the NF-κB-dependent cytokines *in vivo* and *in vitro* (29, 34), but regulation of IL-1β maturation by SLPI has not been investigated yet.

In this study, we discovered a novel anti-inflammatory mechanism, induced by SLPI, which efficiently inhibits ATP-dependent secretion of IL-1β without impairing ATP-independent IL-1β release. We demonstrated that this novel mechanism involves annexin 2 (Anx2), calcium-independent phospholipase A2β (iPLA2β) and the secretion of a small mediator. Our data suggest that this secretory factor may act as a ligand of unconventional nAChRs that in a Src-dependent manner inhibit IL-1β release.

## Materials and Methods

### Animals

All animal experiments were performed following the recommendations of the NIH “Guide for the Care and Use of Laboratory Animals” and were approved by the Regierungspräsidium Giessen, Hesse, Germany (license number 549_M; Gi 20/23-Nr. A12/2011; Gi 20/23-Nr. A10/2011) or by the Regierungspräsidium Karlsruhe, Baden-Württemberg, Germany (license number G248/11). Male and female WT and *Chrna9* (129S-Chrna9tm1Bedv/J), *Chrna10* (129S4-Chrna10tm1Bedv/Mmucd) and *Pla2g6* gene-deficient mice were used. The detailed information about the generation and characterization of the respective gene-deficient mouse strain was reported before (14, 35). *Chrna9* and *Chrna10* gene-deficient mice were kindly provided by Prof. D. E. Vetter, Jackson, MS, USA. *Pla2g6* gene-deficient mice were supplied by Dr. W. Chamulitrat, Heidelberg, Germany. The genotype of every mouse was evaluated by PCR.

### U937 Cells

The human histiocytic lymphoma cell line U937 was purchased from the German Collection of Microorganisms and Cell Cultures (Braunschweig, Germany). The cells were maintained in suspension culture in RPMI 1640 medium (Gibco/Life Technologies, Carlsbad, CA) supplemented with 10% fetal bovine serum (FBS Superior EU, Biochrom GmbH, Berlin, Germany) and 2 mM GlutaMAX^TM^ (Gibco/Life Technologies) at 37°C in a humidified atmosphere of 5% CO_2_. Cells in the log-phase of growth were transferred to 24-well plates (1 x 10^6^ cells/ml and per well) and primed with 1 μg/ml LPS from *Escherichia coli* (L2654; Sigma-Aldrich) for 5 h. Thereafter, 2′(3′)-O-(4-benzoylbenzoyl)adenosine 5′-triphosphate triethylammonium salt BzATP (100 μM; Jena Bioscience, Jena, Germany) or nigericin (50 μM; Sigma-Aldrich) combined with apyrase (0.5 U/ml, Sigma-Aldrich) were applied for 30 min, in the presence or absence of SLPI (0.01 μg−10 μg/ml; R&D Systems, Inc., Minneapolis, MN or provided by Prof. S. Janciaunskiene, Hannover, Germany). To study the involvement of various subunits of nAChRs, the following antagonists were applied: mecamylamine hydrochloride (Mec, 100 μM, Sigma-Aldrich), α-bungarotoxin (α-Bun, 1 μM, Tocris Bioscience, Bristol, UK), strychnine hydrochloride (Stry, 10 μM, Sigma-Aldrich), ArIB [V11L, V16D] (500 nM) or RgIA4 (200 nM). These conopeotides were synthesized as previously described (14, 36, 37). To evaluate the involvement of phospholipase A2 (PLA2), cells were treated with arachidonyl trifluoromethyl ketone (ATK, 50 μM, Enzo Life Science, Lausen, Switzerland) or with bromoenol lactone (BEL, 50 μM, Enzo Life Science). To test the involvement of Src kinase, 4-amino-5-(4-chlorophenyl)-7-(t-butyl)pyrazolo[3,4-d]pyrimidine (PP2, 1-20 μM, Calbiochem, Darmstadt, Germany), a selective inhibitor of Src-family tyrosine kinases, or 4-amino-7-phenylpyrazolo[3,4-d]pyrimidine (PP3, 20 μM, Calbiochem), an inactive analog of the Src tyrosine kinase inhibitor, were applied. Cell culture supernatants were collected and stored at −20°C until IL-1β and lactate dehydrogenase (LDH) measurement.

### Conditioned Media

For the preparation of conditioned media, U937 cells were transferred to a buffered salt solution (containing 5.4 mM KCl, 120 mM NaCl, 2 mM CaCl_2_, 1 mM MgCl_2_, 25 mM glucose, and 10 mM HEPES; pH 7.4) and primed with LPS (1 μg/ml) for 5 h. Thereafter, cells were incubated in the absence (M1) or presence (M2) of SLPI (10 μg/ml) for additional 30 min. Media were harvested, centrifuged (at 500 g for 8 min), and cell-free M1 was supplemented with SLPI (10 mg/ml). Cell-free conditioned media M1 and M2 were ultrafiltrated using Amicon^TM^ Ultra centrifugal filters (Ultracel^TM^ 3K, Merck Millipore, Darmstadt, Germany) with a cut-off of 3 kDa. In some experiments, cells transfected with siRNA were used (M3, M4, M5).

### Mouse Peripheral Blood Mononuclear Cells

Mouse peripheral blood mononuclear cells (mPBMCs) were freshly isolated from heparinized blood obtained from WT, *Chrna9, Chrna10*, or *Pla2g6* gene-deficient mice by Percoll (GE Healthcare Bio-Sciences AB, Uppsala, Sweden) (1.082g/ml) density gradient centrifugation (14). Mononuclear cells were cultured for 2 h in 96-well-plates according to the culture conditions described for U937 cells.

### Human Peripheral Blood Mononuclear Cells

Human peripheral blood mononuclear cells (hPBMCs) were isolated from the heparinized blood of healthy (self-reported) male non-smoking volunteers by density gradient centrifugation using Leucosep gradients (Greiner Bio-One, Frickenhausen, Germany). Before cell isolation, donor blood cells were pulsed with LPS (5 ng/ml). The use of the human blood was approved by the local ethics committee of the University of Giessen (No. 81/13).

### Gene Silencing

The expression of human nAChR subunits α5, α7, α9, α10, iPLA2β, arrestin β1 (Arrb1) arrestin β2 (Arrb2), and Anx2 was down-regulated using siRNA. Cells were transfected with ON-TARGETplus human *CHRNA5* (α5), 7(α7), *CHRNA9* (α9), *CHRNA10* (α10), *PLA2G6* (iPLA2β), *ARRB1* (Arrb1), *ARRB2* (Arrb2) SMARTpool siRNA (Thermo Fisher Scientific, Schwerte, Germany), annexin II siRNA (h2) (Anx2) (Santa Cruz Biotechnology, Dallas, TX) or with ON-TARGETplus non-targeting control pool (con) (Thermo Fisher Scientific) as a negative control. Cells were transfected with 30 pmol siRNA/1 x 10^6^ cells using the Amaxa^TM^ Cell Line Nucleofector Kit^TM^ C and the Nucleofector II Device (both from Lonza, Cologne, Germany) according to the manufacturer's instructions. The siRNA-mediated silencing of target genes was evaluated 24 or 48 h after transfection by real-time RT-PCR or immunoblotting.

### Cloning and Protein Overexpression

Full-length human SLPI cDNA was amplified by PCR using forward primer: 5′-CTC GAG ATG AAG TCC AGC GGC CTC TTC-3′ and reverse primer 5′-GAA TTC TCA AGC TTT CAC AGG GGA AAC-3′ containing a built-in XhoI or EcoRI restriction site, respectively. The PCR product was ligated into the pcDNA3.1 (-) expression vector (Invitrogen, Carlsbad, CA). Identity and accuracy of the construct were confirmed by sequencing. U937 cells were transfected by electroporation with 2 μg of the construct/1 x 10^6^ cells using the Amaxa^TM^ Cell Line Nucleofector Kit^TM^ C and the Nucleofector II Device according to the manufacturer's instructions. The overexpression was evaluated 24 h after transfection by real-time RT-PCR.

### RNA Isolation and cDNA Synthesis

Total RNA was isolated from 1 x 10^6^ U937 cells using the RNeasy Plus^TM^ Mini Kit (Qiagen, Hilden, Germany) according to the manufacturer's instructions. One microgram of RNA was reversely transcribed using M-MLV H- reverse transcriptase and 1 μg of random hexamer primers (both from Promega, Mannheim, Germany).

### Real-Time PCR

Real-time PCR was performed to evaluate the knock-down efficiency of the mRNA of nAChR subunits α5, α7, α9, α10, or iPLA2β after treatment with siRNA. Additionally, the mRNA expression levels of IL-1β and SLPI were determined in different experimental settings. At least 4 samples per experimental group were analyzed; each sample was assessed in duplicates using the ABI 7900 Sequence Detection System (Applied Biosystems, Foster City, CA) and Platinum^TM^ SYBR^TM^ Green qPCR Super Mix-UDG (Invitrogen, Karlsruhe, Germany). The human hydroxymethylbilane synthase (HMBS, synonym PBGD) gene was selected as a reference gene, as it was reported not to be regulated in monocytes under various culture conditions and stimulations (10). Primers specific for the detection of human HMBS, α7, α9, and α10 nAChR subunits, pro-IL-1β and iPLA2β were synthesized by MWG Biotech (Ebersberg, Germany) and their sequences have been published before (13, 38). To analyze SLPI expression, the following primers were used: forward primer: 5′-TCT TCT TCC CTG GAC ACT GC-3′ and reverse primer: 5′-CTG TGG AAG GCT CTG GAA AG-3′. Changes in the mRNA expression levels of the targeted genes were calculated by the 2^ΔCT^ method, where ΔCT represents the difference between the CT value of the HMBS gene and the CT value of the gene of interest. The mean of the mRNA expression values from cells transfected with non-targeting siRNA was set to one and the values from cells transfected with siRNA specific for nAChR subunits or iPLA2β were calculated accordingly.

### Immunoblotting

Immunoblotting was performed essentially as previously described (13). In brief, equal amounts of proteins were separated by SDS-PAGE and transferred to polyvinylidene difluoride (PVDF) membranes (Millipore, Billerica, MA). Membranes were blocked for 1 h with PBS containing 5% low fat milk powder (Roth, Karlsruhe, Germany) followed by overnight incubation at 4°C with primary antibodies anti-IL-1β 1:20,000 (kindly supplied by the National Cancer Institute, Frederick, MD), anti-Anx2 1:1,000 (Abnova, Taipai, Taiwan), anti-Arrb1 1:500 (Zymed, Carlsbad, CA), anti-Arrb2 1:2,000 (Abnova), anti-β-actin 1:100,000 (Sigma-Aldrich) or anti-GAPDH 1:10,000 (Novus Biologicals, Littleton, CO) in blocking solution. After extensive washing, the membranes were further incubated with appropriate horseradish peroxidase (HRP)-conjugated secondary antibodies (Dako, Glostrup, Denmark). For the detection of bound peroxidase, SuperSignal West Dura Extended Duration Substrate (Thermo Fisher Scientific) was used. Only β-actin and GAPDH were revealed with the less sensitive Lumi-Light substrate (Roche, Mannheim, Germany). To quantify the signal, densitometric analyses were performed using a digital gel documentation system (Biozym, Hessisch Oldendorf, Germany).

### Immunocytochemistry

Immunocytochemistry on hPBMCs pulsed with LPS (5 ng/ml; Sigma-Aldrich) and stimulated with ATP (1 mM; Sigma-Aldrich) for 30 min in the presence or absence of SLPI (10 μg/ml) was carried out as previously described (16, 39). Stained cells were evaluated in a blinded manner using an Olympus BX51 microscope and the analySIS software (Olympus, Hamburg, Germany). At least 150 cells in 12 fields of vision were counted per experiment. The total number of cells and specks were indicated as mean number of specks per 100 cells. The number of specks per 100 cells obtained after stimulation with ATP was set to 100% and the remaining values were calculated accordingly.

### ELISA

The concentration of IL-1β in the cell culture supernatants was determined by mouse or human Quantikine Immunoassay^TM^ (R&D Systems, Mineapolis, MN) according to the manufacturer's instructions.

### LDH Measurements

To estimate cell death at the end of each experiment, the release of lactate dehydrogenase (LDH) into the cell culture medium was measured using the Non-Radioactive Cytotoxicity Assay^TM^ (Promega, Madison, WI, USA) according to the manufacturer's instructions. The viability of the cells was not impaired in any condition tested (Supplemental Table S1).

### Two-Electrode Voltage-Clamp Measurements

Whole-cell two-electrode voltage-clamp (TEVC) measurements were performed on cRNA- or water-injected *Xenopus laevis* oocytes, purchased from Ecocyte Bioscience (Castrop-Rauxel, Germany), as previously described (14, 40). The cRNAs encoding for P2X_7_R and α7 nAChR were kindly provided by Prof. G. Schmalzing (Aachen, Germany). The cRNA encoding for human nAChR subunits α9, α10 and Rapsn was synthesized using an *in vitro* transcription kit (T7 RiboMAX™ Large Scale RNA Production System Kit, Promega, Mannheim, Germany). In all experimental groups, measurements were performed on oocytes from at least two different *Xenopus laevis* frogs.

### Whole-Cell Patch-Clamp Recordings

U937 cells were seeded in bath solution (5.4 mM KCl, 20 mM NaCl, 2 mM CaCl_2_, 1 mM MgCl_2_, 10 mM HEPES (4-(2-hydroxyethyl)-piperazine-1-ethanesulfonic acid), 25 mM glucose, pH 7.4) in culture dishes (Nunc, Roskilde, Denmark) and incubated for 5 h with LPS (1 μg/ml) at 37°C. Thereafter, whole-cell recordings were performed at room temperature on an inverted microscope (Axiovert, Zeiss, Göttingen, Germany). Patch pipettes with a resistance of 2–4 MΩ were pulled from borosilicate glass capillaries (Hilgenberg, Malsfeld, Germany) using an automated puller (Zeitz, Augsburg, Germany) and filled with pipette solution (120 mM KCl, 1 mM, CaCl_2_, 2 mM MgCl_2_, 10 mM HEPES, 11 mM ethylene glycol tetraacetic acid, 20 mM glucose, pH 7.39). The whole-cell recordings were performed at a voltage-clamped membrane potential of −60 mV, and transmembrane currents were amplified with an EPC 9 amplifier (HEKA, Lambrecht, Germany) and acquired via an ITC-16 interface (HEKA). ATP (1 mM; Sigma) and SLPI (10 μg/ml) were dissolved in bath solution and applied via a pressure-driven microperfusion system.

### Statistical Analysis and Data Processing

Data were analyzed using SPSS software (IBM, Munich, Germany) by Wilcoxon signed-rank test or by the non-parametric Kruskal-Wallis test, followed by the Mann-Whitney rank-sum test. A *p*-value below 0.05 was considered as statistically significant and marked with ^*^. Data are presented as individual data points, bar represents median whiskers percentiles 25 and 75. The number of independent experiments (at least 4 per group) is indicated in the figures.

## Results

### The BzATP-Mediated Release of IL-1β by Monocytic U937 Cells Is Inhibited by SLPI

**Figure 1:**
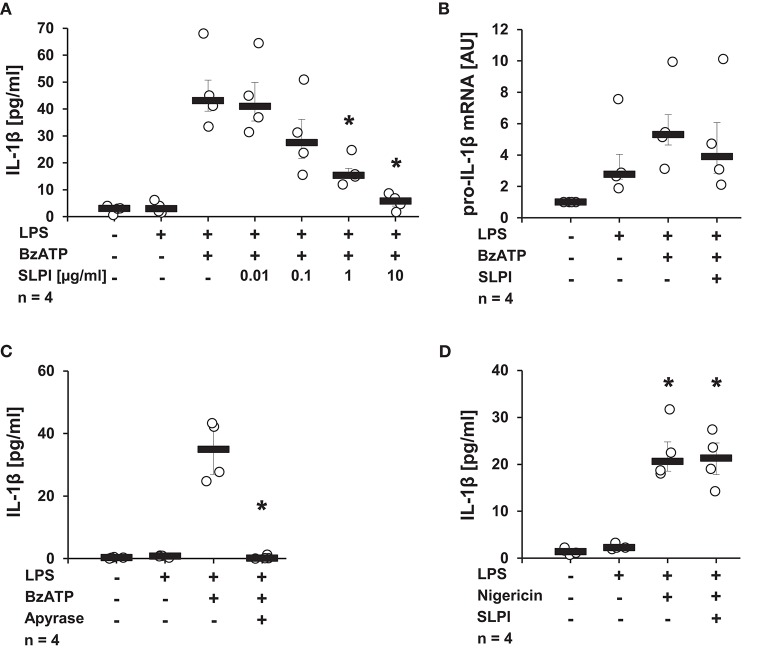
SLPI inhibits BzATP-mediated IL-1β release by U937 cells. Human monocytic U937 cells were primed with LPS (1 μg/ml, for 5 h) and further stimulated with 2′(3′)-O-(4-benzoylbenzoyl)adenosine 5′-triphosphate triethylammonium salt (BzATP; 100 mM) or nigericin (50 μM) for 30 min. The concentration of IL-1β in cell culture supernatants was measured by ELISA. The mRNA expression of pro-IL-1β was assessed by real-time PCR. **(A)** SLPI dose-dependently and effectively inhibited the BzATP-mediated IL-1β release. **(B)** The LPS-induced mRNA levels of pro-IL-1β were not changed upon stimulation with SLPI. AU, arbitrary units **(C)** The treatment with apyrase (0.5 U/ml) fully abolished the BzATP-mediated IL-1β release. **(D)** The nigericin-mediated IL-1β secretion was not affected in the presence of SLPI. ^*^p ≤ 0.05 significantly different compared to cells stimulated with LPS and BzATP **(A,C)** or LPS alone **(D)**. Experimental groups were compared by Kruskal-Wallis test followed by Mann-Whitney rank sum test.

Initial experiments were performed to determine, whether SLPI can modulate IL-1β release. Human monocytic U937 cells were primed for 5 h with LPS (1 μg/ml) to induce pro-IL-1β expression, followed by stimulation with the P2X_7_R agonist, BzATP (100 μM) for additional 30 min in the presence or absence of varying concentrations of SLPI (0.01 μg−10 μg/ml). IL-1β levels in the cell culture supernatant were quantified by ELISA. In agreement with previous reports, the treatment with BzATP induced the release of relatively low amounts of IL-1β (10, 14, 38, 39). SLPI inhibited the BzATP-mediated secretion of IL-1β in a dose-dependent manner with an IC_50_ of 0.26 μg/ml (Figure 1A). To test if the mRNA expression of pro-IL-1β is directly affected by SLPI, real-time PCR was performed. As expected, LPS significantly increased pro-IL-1β mRNA expression levels compared with untreated control cells. In contrast, no significant changes in pro-IL-1β mRNA levels could be seen upon application of SLPI (Figure 1B). As an additional control, the ATP-degrading enzyme apyrase (0.5 U/ml) was applied together with BzATP. Apyrase completely abrogated IL-1β secretion (Figure 1C). Furthermore, the BzATP-independent IL-1β release mediated via the pore-forming bacterial toxin nigericin was investigated. In this experimental setting LPS-primed U937 cells were stimulated with nigericin (50 μM) for 30 min. To ensure the absence of ATP that could be released by U937 cells, apyrase (0.5 U/ml) was additionally applied. As previously reported, nigericin induced IL-1β secretion (13, 38), but the IL-1β release was not inhibited in the presence of SLPI (10 μg/ml) (Figure 1D). Cell death, as measured by the detection of LDH activity in the cell culture supernatants, was not increased in these and any of the following experiments (Supplemental Table S1).

### The BzATP-Mediated Release of IL-1β by hPBMCs Is Inhibited by SLPI

**Figure 2:**
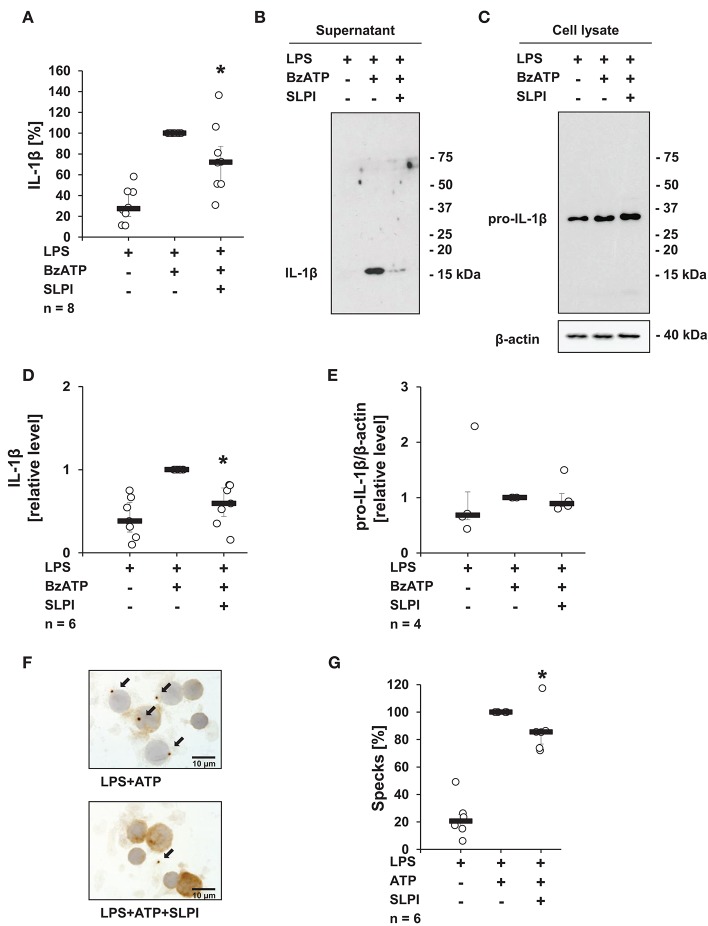
SLPI inhibits the BzATP-induced IL-1β release by human peripheral blood mononuclear cells (hPBMCs). Blood from healthy volunteers was pulsed with LPS (0.5 ng/ml) before isolation of mononuclear cells. Purified hPBMCs were cultured for 3 h and stimulated for 30 min with 2′(3′)-O-(4-benzoylbenzoyl)adenosine 5′-triphosphate triethylammonium salt (BzATP, 100 μM) in the presence or absence of SLPI (10 μg/ml). The concentration of IL-1β in cell culture supernatants was measured by ELISA or assessed by immunoblotting. **(A)** SLPI inhibited the BzATP-mediated IL-1β release. The amount of IL-1β released upon stimulation with BzATP was set to 100% and the remaining values were calculated accordingly. ^*^p ≤ 0.05 significantly different compared to cells stimulated with LPS and BzATP. Experimental groups were compared by Wilcoxon signed-rank test. **(B)** The level of IL-1β detected in cell culture supernatants was diminished upon treatment with SLPI; one representative immunoblot out of 6 is shown. **(C)** Equal levels of pro-IL-1β were detected by immunoblotting in cell lysates obtained from hPBMCs independent of the treatment. Detection of β-actin served as a loading control. One representative blot out of 4 is shown. **(D)** Optical density of the immune-positive bands was measured. The values obtained after LPS and BzATP stimulation were set to one and the rest of the values were calculated accordingly. **(E)** Optical density of the immune-positive bands for pro-IL-1β was measured and divided by the values obtained for β-actin on the same blot. The values obtained after LPS and BzATP stimulation were set to one, all other values were calculated accordingly. ^*^p ≤ 0.05 significantly different compared to cells stimulated with LPS and BzATP **(D,E)**. Experimental groups were compared by Kruskal-Wallis test followed by Mann-Whitney rank sum test. **(F)** ASC (apoptosis-associated speck-like protein containing a caspase activation and recruitment domain) specks (arrows) were detected by immunocytochemistry using antibodies directed to ASC (brown staining). Cell nuclei were delicately counterstained with hemalum. Representative specimens out of 6 individual experiments each are shown. **(G)** Specks were induced by the treatment with ATP (1 mM) and significantly reduced, when SLPI was given concomitantly. The number of specks per 100 cells obtained after stimulation with ATP was set to 100% and the remaining values were calculated accordingly. ^*^p ≤ 0.05 significantly different compared to cells pulsed with LPS and stimulated with ATP **(G)**. Experimental groups were compared by Wilcoxon signed-rank test.

To test, if the inhibitory mechanism mediated by SLPI also applies to primary cells, hPBMCs isolated from the blood of healthy volunteers were examined. In agreement with previously published results (13, 38), adherent hPBMCs pulsed with LPS (5 ng/ml), secreted small amounts of IL-1β (median 449 pg/ml), which was increased upon stimulation with BzATP (median 2,238 pg/ml). As expected, in the presence of SLPI (10 μg/ml) the BzATP-mediated IL-1β release was significantly lowered (median 1,370 pg/ml) (Figure 2A). However, under the same experimental conditions hPBMCs isolated from two out of eight healthy donors did not respond to SLPI. ELISA data were confirmed by immunoblotting combined with densitometric quantification of the signal (Figures 2B,D). Of note, cell culture supernatant was devoid of pro-IL-1β. Pro-IL-1β was detected by immunoblotting only in cell lysates of hPBMCs and regardless of the treatment with BzATP or SLPI, there seemed to be no difference in the intensity of the signal (Figures 2C,E). Only one out of four cell lysates showed an immune positive band in the molecular mass range of mature IL-1β. To confirm our findings, the formation of so-called ASC (apoptosis-associated speck-like protein containing a caspase activation and recruitment domain) specks was investigated by immunocytochemistry using antibodies directed to ASC. The number of ASC specks observed in LPS-pulsed hPBMCs stimulated with ATP (1 mM) seemed to be lower in the presence of SLPI (10 μg/ml) but did not reach statistical significance (*p* = 0.116) (Figures 2F,G).

### The BzATP-Mediated Secretion of IL-1β Is Reduced Upon Overexpression of SLPI

**Figure 3:**
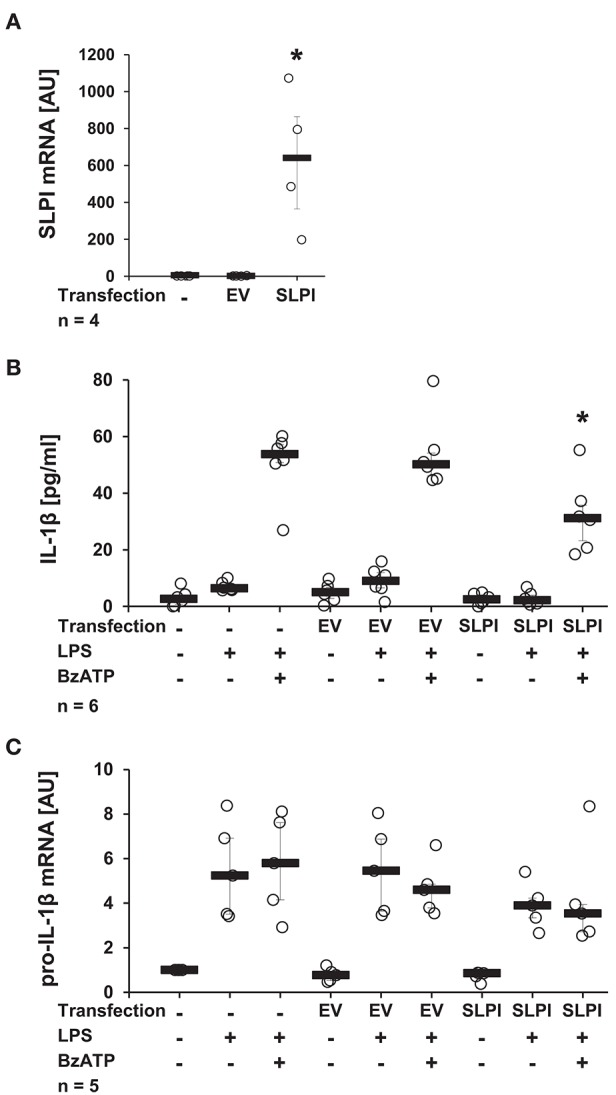
Overexpression of SLPI reduces the BzATP-mediated IL-1β release by U937 cells. Human monocytic U937 cells were left untreated, transfected with the empty vector (EV), or with an expression vector containing the coding sequence for human SLPI (SLPI). Twenty-four hours after transfection, the cells were primed with LPS (1 μg/ml, for 5 h) and further stimulated with 2′(3′)-O-(4-benzoylbenzoyl)adenosine 5′-triphosphate triethylammonium salt (BzATP; 100 mM) for 30 min. The concentration of IL-1β in cell culture supernatants was measured by ELISA. The mRNA expression of SLPI or pro-IL-1β was assessed by real-time PCR. **(A)** Transfection with SLPI expression vector increased the mRNA level of SLPI compared to cells transfected with EV. **(B)** The BzATP-induced IL-1β release was significantly lower in cells overexpressing SLPI compared to cells transfected with EV. **(C)** The mRNA level of pro-IL-1β was not affected by SLPI overexpression; ^*^p ≤ 0.05 significantly different compared to cells transfected with EV **(A)** or transfected with EV and stimulated with LPS and BzATP **(B,C)**. Experimental groups were compared by Kruskal-Wallis test followed by Mann-Whitney rank sum test.

It has been well-documented that monocytes/macrophages are able of expressing and secreting SLPI (38). In the following approach, we investigated if elevated endogenous levels of SLPI modulate the BzATP-mediated IL-1β release by monocytes. U937 cells were transfected with a pcDNA expression vector containing the coding sequence of human SLPI. To ensure effective overexpression, the mRNA levels of SLPI were determined 24 h after transfection. As predicted, the mRNA expression levels of SLPI were significantly elevated compared to cells transfected with empty vector (EV) (Figure 3A). Overexpression of SLPI significantly lowered the BzATP-induced IL-1β release (Figure 3B), whereas the level of pro-IL-1β mRNA were unaffected by SLPI overexpression (Figure 3C).

### The Ion Channel Function of Heterologously Expressed P2X_7_R Is not Modulated by SLPI

**Figure 4:**
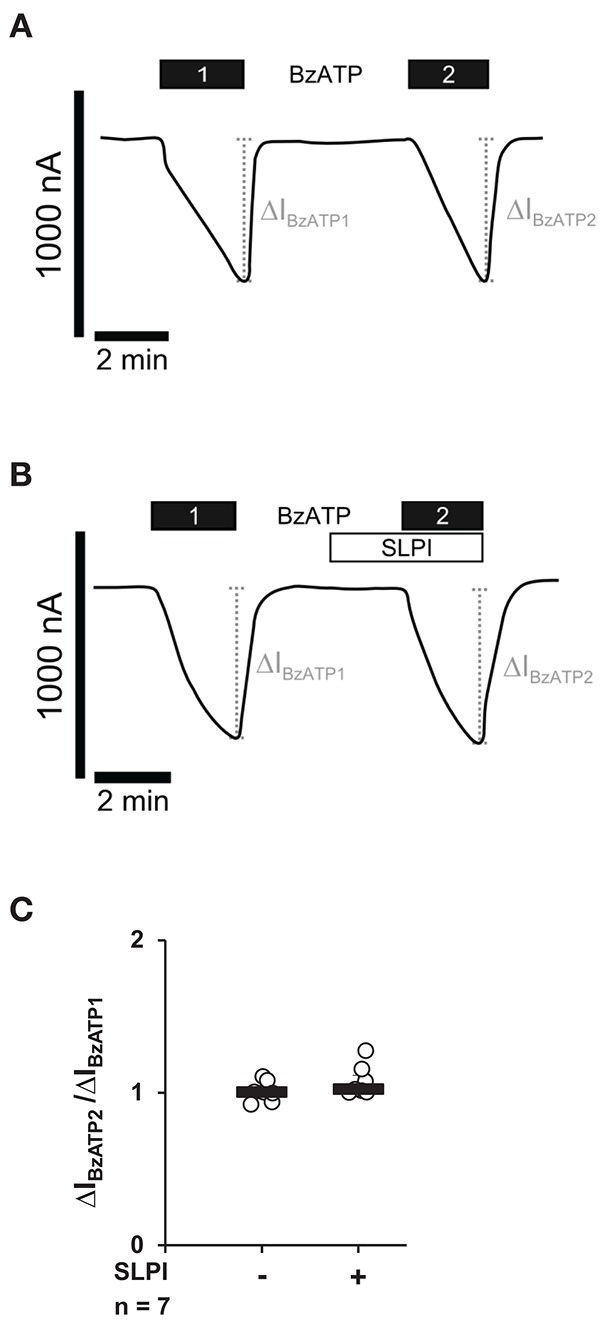
SLPI does not directly modulate the ion channel function of heterologously expressed P2X_7_ receptors (P2X_7_Rs). Whole-cell two-electrode voltage-clamp measurements (TEVC) were performed on *Xenopus laevis* oocytes that heterologously expressed human P2X_7_Rs. **(A)** Stimulation with 2′(3′)-O-(4-benzoylbenzoyl)adenosine 5′-triphosphate triethylammonium salt (BzATP; 10 μM) induced repetitive changes in transmembrane ion currents (ΔI_BzATP1_ and ΔI_BzATP2_). **(B)** Application of SLPI (10 μg/ml) had no impact on ΔI_M_ and did not impair the consecutive response to BzATP. **(A,B)** Representative current curves are shown. **(C)** Graphical representation of normalized ΔI_BzATP2_/ΔI_BzATP1_ are shown **(A,B)**. Experimental groups were compared by Wilcoxon signed-rank test.

Next, we investigated if SLPI directly impairs the ion channel function of P2X_7_R. *Xenopus laevis* oocytes were injected with cRNA encoding the human P2X_7_R, and TEVC measurements were performed. As expected (37), stimulation of heterologously P2X_7_R expressing *Xenopus* oocytes with BzATP (10 μM) evoked transmembrane ion currents (ΔI_M_) that were reversible by washout and repeatable by subsequent application of BzATP (Figures 4A,C). The BzATP-induced currents were unimpaired in the presence of SLPI (10 μg/ml) (Figures 4B,C).

### SLPI Inhibits the Function of Monocytic ATP Receptors

**Figure 5:**
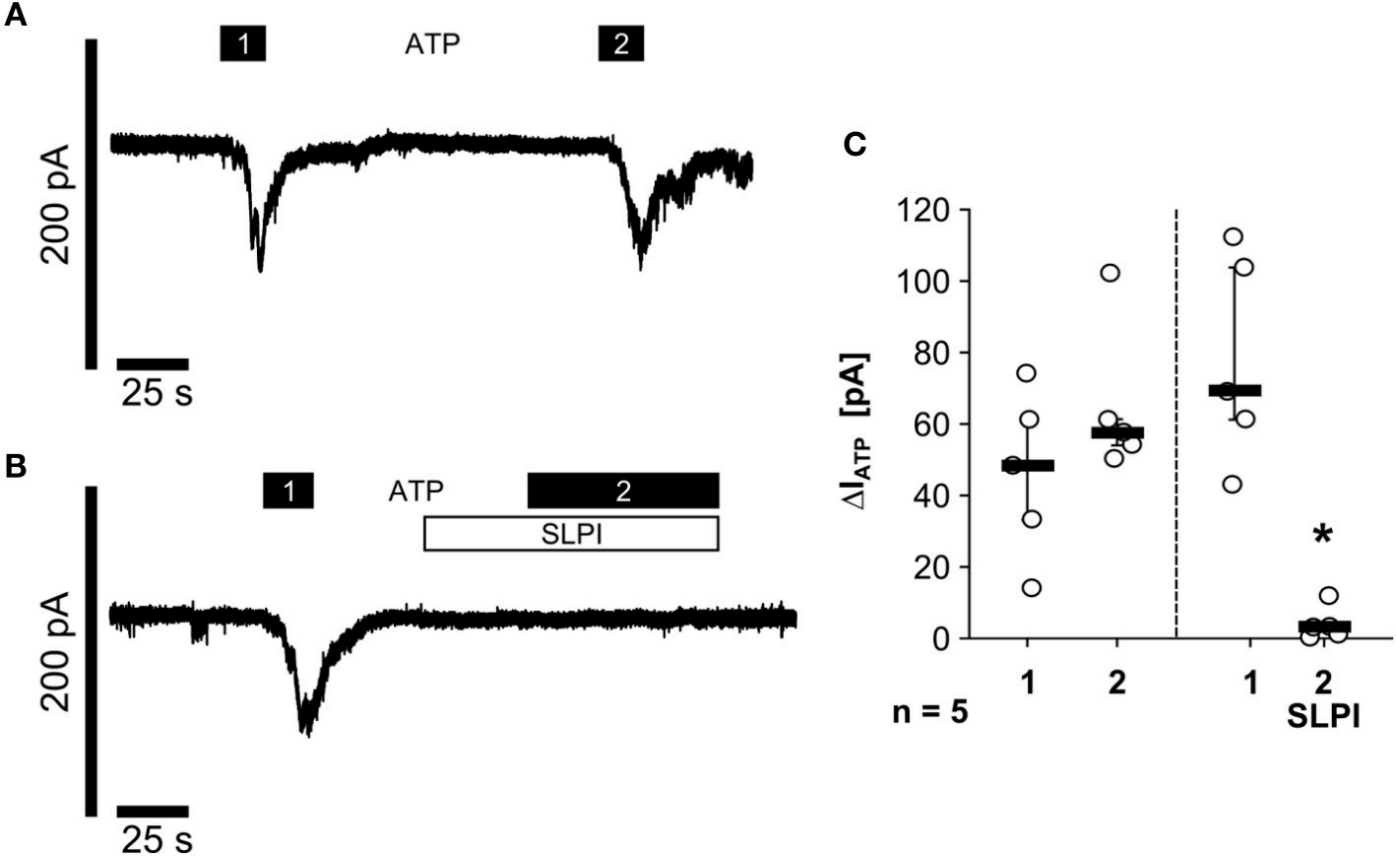
SLPI inhibits the function of monocytic ATP receptors. Human monocytic U937 cells were primed with LPS (1 μg/ml, for 5 h). Whole-cell patch-clamp recordings were performed and ion-current changes in response to two applications (1 and 2) of ATP (1 mM) were recorded in the absence **(A,C)** or presence **(B,C)** of SLPI (10 μg/ml). **(C)** All ATP-induced current changes (ΔI_ATP_) are presented as individual data points, bars indicate median, whiskers percentiles 25 and 75, experimental groups were compared by Wilcoxon signed-rank test; ^*^p ≤ 0.05 significantly different compared to the first ΔI_ATP_.

As nigericin-induced IL-1β release was not sensitive to SLPI (Figure 1D), it was unlikely that SLPI inhibits the formation of the NLRP3 inflammasome or caspase-1 activation. Hence, we performed whole-cell patch-clamp experiments on U937 cells to test if SLPI application results in an impaired function of monocytic ATP receptors. Application of ATP (1 mM) induced repeatable ion-current changes that were almost fully inhibited by SLPI (10 μg/ml) (Figures 5A–C). These results together with those shown in Figure 4 suggest that SLPI induces a signaling cascade in monocytic cells that results in an indirect inhibition of P2X_7_Rs.

### The nAChR Subunits α7, α9, and α10 Are Mandatory for the Function of SLPI

**Figure 6:**
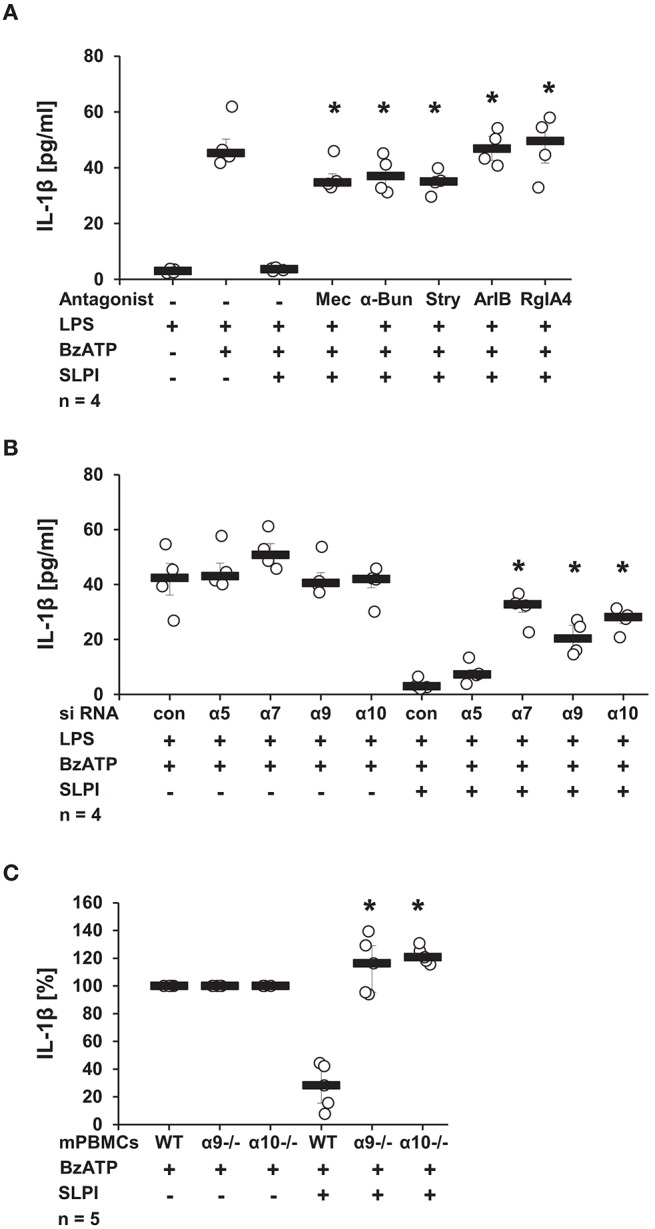
SLPI signaling involves nicotinic acetylcholine receptor (nAChR) subunits α7, α9, and α10. Human monocytic U937 cells were primed with LPS (1 μg/ml, for 5 h) and further stimulated with 2′(3′)-O-(4-benzoylbenzoyl)adenosine 5′-triphosphate triethylammonium salt (BzATP; 100 mM) for 30 min. **(A)** Application of nicotinic antagonists mecamylamin (Mec), α-bungarotoxin (α-Bun), strychnine (Stry), RgIA4, or ArIB abolished the SLPI-mediated inhibition of BzATP-induced IL-1β release; ^*^p ≤ 0.05 significantly different compared to cells stimulated with LPS, BzATP, and SLPI. **(B)** Down-regulation of the expression of nAChR subunits α7, α9, or α10 by siRNA blunted the SLPI-induced inhibitory mechanism. ^*^p ≤ 0.05 significantly different compared to cells transfected with control siRNA (con) and stimulated with LPS, BzATP and SLPI. **(C)** Mouse peripheral blood mononuclear cells (mPBMCs) deficient in α9 or α10 nAChR subunits stimulated with BzATP were resistant to the SLPI-mediated inhibition. The amount of IL-1β released upon stimulation with BzATP was set to 100% and the remaining values were calculated accordingly. ^*^p ≤ 0.05 significantly different compared to mPBMCs obtained from wild-type (WT) mice stimulated with BzATP and SLPI. Experimental groups were compared by Kruskal-Wallis test followed by Mann-Whitney rank sum test.

Recently, a novel cholinergic mechanism that inhibits BzATP-mediated current changes and, hence, the release of IL-1β via nAChRs composed of subunits α7, α9, and α10 was reported by our group (13–15). To test if nAChRs are also necessary for the SLPI-mediated inhibition of IL-1β secretion, a panel of nAChR antagonists was applied. The presence of Mec, a known general inhibitor of nAChRs (41), completely abolished the inhibitory function of SLPI (Figure 6A). Similarly, α-Bun and Stry, two nAChR antagonists that preferentially inhibit nAChR subunits α7 and α9 (42, 43), effectively abrogated the inhibitory effect mediated by SLPI (Figure 6A). In line with these observations, treatment with ArIB [V11L, V16D] or RgIA4 reported as selective antagonists of nAChRs containing subunits α7 or α9/α10, respectively (36, 37), blocked the inhibitory function of SLPI (Figure 6A).

To further confirm the importance of nAChR subunits α7, α9, and α10 in the inhibitory mechanism mediated by SLPI, the expression of these nAChR subunits in U937 cells was down-regulated using siRNA. The efficiency and specificity of the single knock-down was recently positively evaluated at the mRNA level in the same experimental setting (13, 14). In agreement with results obtained using nAChR antagonists, SLPI was able to effectively inhibit the BzATP-mediated IL-1β release from cells transfected with control, non-target siRNA or siRNA specific for the nAChR subunit α5, whereas down-regulation of the expression of nAChR subunits α7, α9, or α10 significantly blunted the inhibitory function of SLPI (Figure 6B). To ensure the importance of nAChR subunits α9 and α10, additionally, mPBMC isolated from WT mice and mice deficient in single nAChR subunit genes were tested. In response to the stimulation with BzATP, mPBMCs from WT mice released IL-1β in to the culture medium (median 39 pg/ml). PBMCs from WT mice released IL-1β in to the culture medium in response to the stimulation with BzATP (median 39 pg/ml). As predicted, SLPI significantly inhibited the secretion of IL-1β from WT mPBMCs (median 9 pg/ml). In contrast, the IL-1β release from mPBMCs deficient in *Chrna9* (α9) (median 129 pg/ml in the absence of SLPI; 166 pg/ml in the presence of SLPI) or *Chrna10* (α10) (median 65 pg/ml in the absence of SLPI; 83 pg/ml in the presence of SLPI) remained unchanged upon treatment with SLPI (Figure 6C).

### SLPI does Not Induce Ion Channel Functions at Heterologously Expressed nAChRs

**Figure 7:**
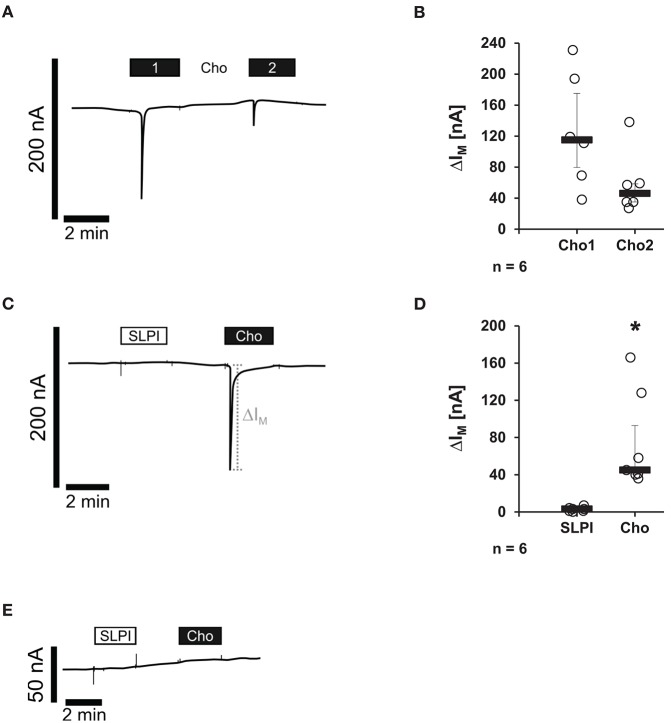
SLPI does not induce ion channel functions at oocytes co-expressing nicotinic acetylcholine receptor (nAChR) subunits α7, α9, and α10. Two-electrode voltage-clamp measurements (TEVC) were performed on *Xenopus laevis* oocytes that heterologously co-expressed human nAChR subunits α7, α9, and α10. **(A–D)** Choline (Cho) induced repeatable current responses, whereas initial SLPI application had no impact on ion currents. **(A,C)** Representative current curves are shown. **(C,D)** Graphical representation of the results of experiments shown in **(B,D)**. **(E)** In water-injected control oocytes, neither SLPI nor Cho application induced any current response. ^*^p ≤ 0.05 significantly different compared to cells stimulated with SLPI. Experimental groups were compared by Wilcoxon signed-rank test.

Binding of canonical nicotinic agonists has been well-documented to induce ion channel functions of nAChRs (10). Therefore, potential changes in the ion currents of these receptors were tested in the presence of SLPI (10 μg/ml). *Xenopus laevis* oocytes were co-injected with cRNA encoding human α7, α9, and α10 nAChR subunits and TEVC measurements were performed. To control for the expression of nAChRs, Cho (1 mM) was applied as a positive control. As expected (15), Cho evoked ion currents that were reversible by Cho washout and repeatable by subsequent Cho application (Figures 7A,B). In contrast, SLPI did not induce any ion currents, whereas Cho did (Figures 7C,D). Neither SLPI nor Cho induced changes in ion currents of control oocytes that were injected with water instead of cRNA (Figure 7E).

### The Inhibitory Mechanism Mediated by SLPI Depends on Src Kinase Activation and Arrb Expression

**Figure 8:**
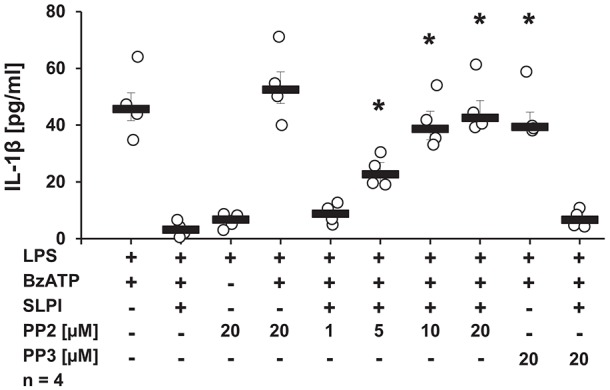
SLPI signals via Src kinase. Human monocytic U937 cells were primed with LPS (1 μg/ml, for 5 h) and further stimulated with 2′(3′)-O-(4-benzoylbenzoyl)adenosine 5′-triphosphate triethylammonium salt (BzATP; 100 mM) for 30 min. SLPI was applied in the presence of different concentrations of the Src kinase inhibitor (PP2) or its inactive analogue (PP3). The concentration of IL-1β in cell culture supernatants was measured by ELISA. PP2 suppressed the SLPI-mediated inhibition. ^*^p ≤ 0.05 significantly different compared to cells stimulated with LPS, BzATP, and SLPI. Experimental groups were compared by Kruskal-Wallis test followed by Mann-Whitney rank sum test.

Since stimulation with SLPI did not induce ion channel functions at heterologously expressed nAChRs, it is plausible that nAChRs exerts metabotropic functions in response to SLPI. There are several downstream molecules described to physically interact with nAChRs and to be involved in the cholinergic signaling (43–46). Initially, the involvement of Src kinase in the inhibitory mechanism mediated by SLPI was examined. LPS-primed U937 cells were stimulated with BzATP and SLPI in the presence of different concentrations of a selective inhibitor of the Src-family tyrosine kinases PP2. Application of PP2 dose-dependently abolished the inhibitory effect mediated by SLPI (Figure 8). As a control, PP3, an inactive analog of the Src tyrosine kinase inhibitor was used. As awaited, the SLPI-mediated inhibitory mechanism remained fully functional in the presence of PP3 (Figure 8). Neither PP2 nor PP3 changed the BzATP-induced release of IL-1β in the absence of SLPI (Figure 8).

**Figure 9:**
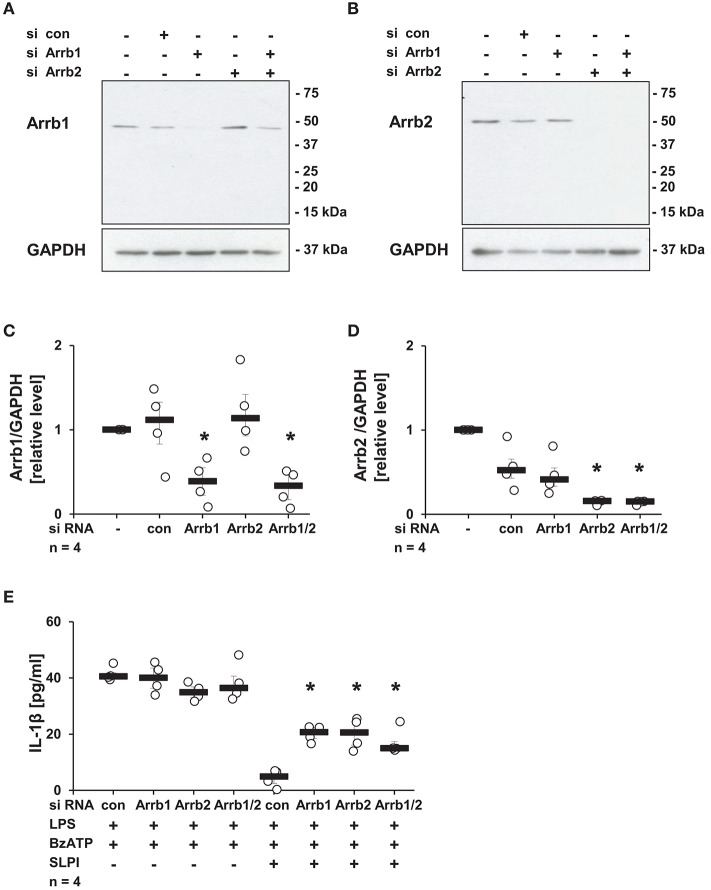
SLPI signaling involves arrestin β1 and 2 (Arrb1/2). Expression of Arrb1 or Arrb2 in U937 cells was diminished using siRNA. Afterwards, cells were primed with LPS (1 μg/ml, for 5 h) and further stimulated with 2′(3′)-O-(4-benzoylbenzoyl)adenosine 5′-triphosphate triethylammonium salt (BzATP; 100 mM) for 30 min in the presence or absence of SLPI (10 μg/ml). The efficiency and specificity of the knock-down was confirmed by immunoblotting. The concentration of IL-1β in cell culture supernatants was measured by ELISA. **(A,C)** The expression level of Arrb1 was selectively lowered upon transfection with Arrb1 siRNA; **(A)** representative immunoblot out of 4 **(B,D)** The expression level of Arrb2 was diminished upon transfection with Arrb2 siRNA; **(B)** representative immunoblot out of 4. **(C,D)** Optical density of the immune-positive bands for Arrb1 or Arrb2 was measured and divided by the values obtained for β-actin on the same blot. The values gathered from untreated cells were set to one and all other values were calculated accordingly. ^*^p ≤ 0.05 significantly different compared to cells transfected with control (con) siRNA. **(E)** Down-regulation of the expression of Arrb1 or Arrb2 by siRNA blunted the SLPI-induced inhibitory effect. ^*^p ≤ 0.05 significantly different compared to cells transfected with control siRNA (con) and stimulated with LPS, BzATP and SLPI. Experimental groups were compared by Kruskal-Wallis test followed by Mann-Whitney rank sum test.

Subsequently, the requirement of Arrb1 or Arrb2, known scaffolding proteins important in the recruitment of Src kinases to nAChRs (46), was examined. Expression of Arrb1 or Arrb2 was selectively reduced using siRNA. The efficiency and specificity of Arrb1 and Arrb2 down-regulation were confirmed by immunoblotting (Figures 9A,B) followed by densitometry (Figures 9C,D). SLPI effectively inhibited the BzATP-mediated IL-1β release from cells transfected with control siRNA. In contrast, down-regulation of the expression of Arrb1 or Arrb2 alone or in combination significantly blunted the inhibitory function of SLPI (Figure 9E).

### Activation of iPLA2β Is Involved in Signaling of SLPI

**Figure 10:**
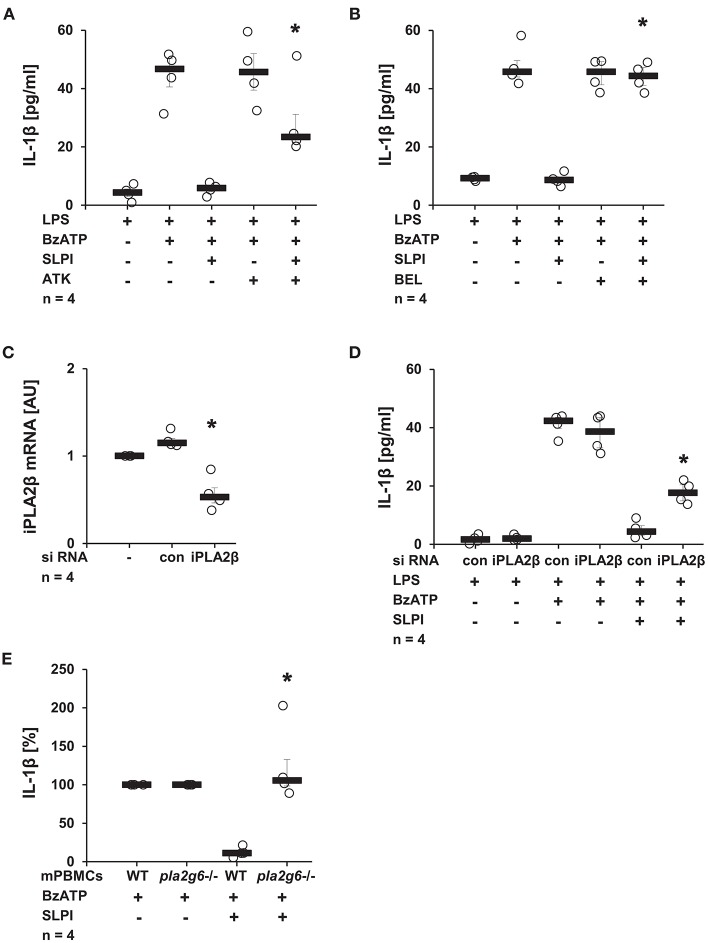
SLPI activates calcium-independent phospholipase A2β (iPLA2β). Human monocytic U937 cells were primed with LPS (1 μg/ml) for 5 h and further stimulated with 2′(3′)-O-(4-benzoylbenzoyl)adenosine 5′-triphosphate triethylammonium salt (BzATP; 100 mM) for 30 min. SLPI was applied in the presence of different PLA2 inhibitors. The concentration of IL-1β in cell culture supernatants was measured by ELISA. **(A)** Treatment with the general PLA2 inhibitor arachidonyl trifluoromethyl ketone (ATK) or **(B)** application of bromoenol lactone (BEL), more specific for iPLA2β, blunted the SLPI-mediated inhibition. **(A,B)** *n* = 4, ^*^p ≤ 0.05 significantly different compared to cells stimulated with LPS, BzATP, and SLPI. **(C,D)** The expression of iPLA2β was silenced by siRNA. **(C)** The mRNA level of iPLA2β, as measured by real-time PCR, was diminished upon transfection with iPLA2β siRNA. ^*^p ≤ 0.05 significantly different compared to cells transfected with control siRNA (con) **(D)** Silencing of the iPLA2β expression diminished the SLPI-mediated inhibitory effect on the BzATP-induced release of IL-1β. *n* = 4, ^*^p ≤ 0.05 significantly different compared to cells transfected with control siRNA (con) and stimulated with LPS, BzATP and SLPI. **(E)** Mouse peripheral blood mononuclear cells (mPBMCs) isolated from wild-type (WT) or *Pla2g6*-gene-deficient mice were stimulated with BzATP in the presence and absence of SLPI (10 μg/ml). The IL-1β released to cell culture supernatants was measured by ELISA. The IL-1β concentration in the absence of SLPI was set to 100% and values after SLPI application were calculated accordingly. ^*^p ≤ 0.05 significantly different compared to cells from WT mice stimulated with BzATP and SLPI. Experimental groups were compared by Kruskal-Wallis test followed by Mann-Whitney rank sum test.

Recently, our group reported the involvement of iPLA2β in chemokine-, β-nicotinamide adenine dinucleotide- and α1-antitrypsin-mediated mechanisms controlling the BzATP-induced IL-1β release by monocytic cells (10, 38, 47). To test the importance of this enzyme in the SLPI-mediated regulation of IL-1β secretion, U937 cells were primed with LPS for 5 h and stimulated for additional 30 min with BzATP and SLPI in the presence and absence of PLA2 inhibitors. ATK, a general inhibitor of PLA2 (48, 49), significantly diminished the inhibitory effect mediated by SLPI (Figure 10A). The presence of BEL, an inhibitor that is more specific for iPLA2β (48), fully abolished the effect of SLPI (Figure 10B). None of these inhibitors changed the amount of IL-1β secreted upon stimulation with BzATP in the absence of SLPI (Figures 10A,B). To further corroborate the importance of iPLA2β, two independent approaches were pursued. First, the iPLA2β expression in U937 cells was silenced using siRNA. The efficiency of this knock-down was evaluated on the mRNA level using real-time PCR (Figure 10C). Furthermore, protein levels were shown to be down-modulated by transfection of specific siRNA in the same experimental setting and the results were recently published (38). As expected, the IL-1β release from U937 cells transfected with control non-target siRNA was fully inhibited upon stimulation with SLPI, whereas the inhibition was significantly blunted in cells treated with siRNA specific for iPLA2β (Figure 10D). The down-regulation of iPLA2β expression did not affect the BzATP-mediated IL-1β secretion in the absence of SLPI. In line with these observations, the IL-1β secretion by mPBMCs isolated from the blood of *pla2g6* gene-deficient mice was not altered upon stimulation with SLPI, whereas the inhibitory mechanism was fully functional in cells obtained from WT animals (Figure 10E). PBMCs from WT mice secreted 49 pg/ml (median) IL-1β in response to BzATP and 7 pg/ml in the presence of BzATP plus SLPI. In supernatants of BzATP-stimulated mPBMCs from mice deficient in *pla2g6*, independent of the presence or absence of SLPI, measured levels of IL-1β were around 100 pg/ml.

### SLPI Induces the Release of a Bioactive Low Molecular Mass Factor

**Figure 11:**
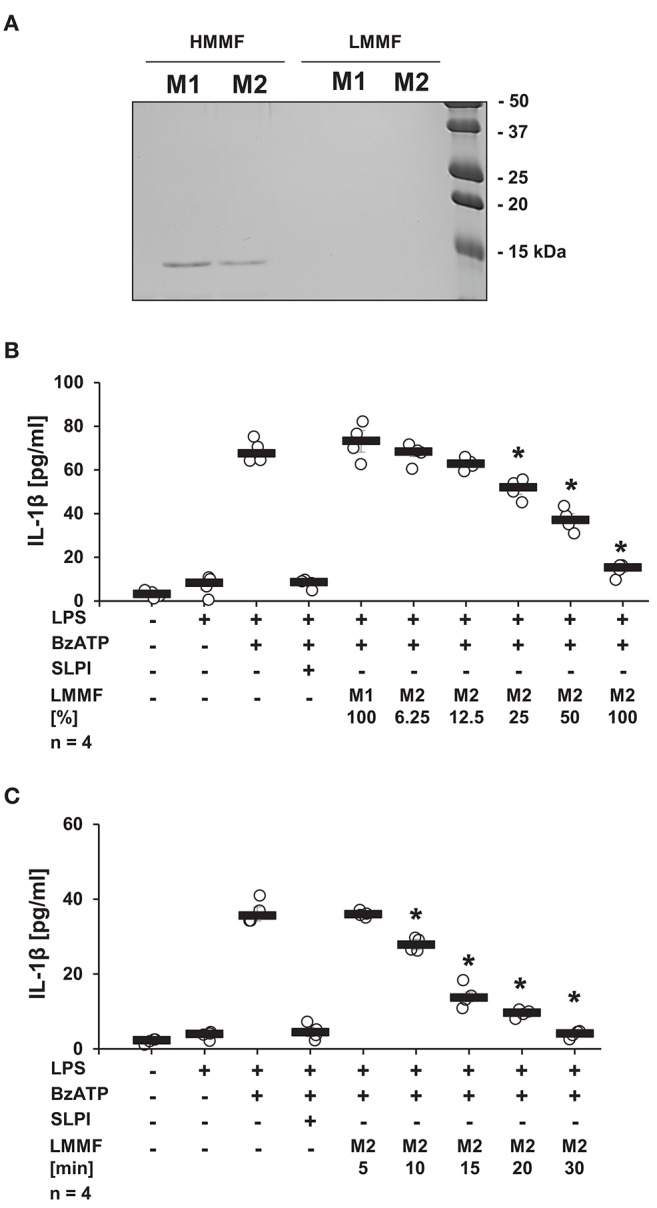
SLPI induces the release of a bioactive factor. Human monocytic U937 cells were primed with LPS (1 μg/ml) for 5 h and further cultured for additional 30 min in the absence (M1) and presence (M2) of SLPI (10 μg/ml). Conditioned media were collected and separated by ultrafiltration (cut-off 3 kDa) into high (HMMF) and low molecular mass fractions (LMMF). Conditioned medium M1 was supplemented with SLPI shortly before ultrafiltration. **(A)** HMMF and LMMF of M1 and M2 were separated by SDS-PAGE (15% acrylamide) along with marker proteins and stained with Coomassie Brilliant Blue. A band with an apparent molecular mass of about 15 kDa corresponding to SLPI was only detected in the HMMF **(B)** LPS-primed U937 cells were stimulated with 2′(3′)-O-(4-benzoylbenzoyl)adenosine 5′-triphosphate triethylammonium salt (BzATP; 100 mM) for 30 min in the presence of different concentrations of the LMMF of M2. LMMF of M1 was applied as a control. LMMF of M2 dose-dependently inhibited the BzATP-induced IL-1β release. **(C)** LMMF of M2 collected at different time-points after SLPI application was applied to U937 cells primed with LPS and stimulated with BzATP for 30 min. The IL-1β release was measured by ELISA. *n* = 4, ^*^p ≤ 0.05 significantly different compared to U937 cells stimulated with LPS and BzATP. Experimental groups were compared by Kruskal-Wallis test followed by Mann-Whitney rank sum test.

It is well-documented that activated iPLA2β catalyzes the release of free fatty acids from various phospholipids (50). Free fatty acids can be further converted into diverse physiologically active lipid mediators. Cleavage of phosphatidylcholines, additionally leads to the generation of PC-containing metabolites with immunonomodulatory functions (51). To test whether stimulation with SLPI results in the production and release of bioactive factors, conditioned medium was produced and tested. LPS-primed U937 cells were stimulated with SLPI (10 μg/ml) for 30 min, the cell-free supernatant was collected and ultrafiltrated at a cut-off of 3 kDa to remove SLPI (M2). Control supernatant was harvested from LPS-primed U937 cells and SLPI was added to the cell-free supernatant shortly before ultrafiltration (M1). To test if SLPI was efficiently separated from the conditioned medium, low and high molecular mass fractions of the conditioned medium M1 and M2 were separated by SDS-PAGE and proteins were visualized with Coomassie Brilliant Blue staining. As predicted, a signal corresponding to the molecular mass of SLPI was only detected in the high molecular mass fractions of conditioned medium (Figure 11A). Low molecular mass fractions were applied to another set of LPS-primed U937 cells together with BzATP and the release of IL-1β was measured 30 min later. The low molecular mass fraction of the M2 supernatant dose-dependently inhibited the BzATP-induced IL-1β release (Figure 11B), whereas the low molecular mass fraction of the M1 supernatant had no effect (Figure 11B). To study the time course of the release of the inhibitory factor, conditioned media M2 were collected every 5 min after application of SLPI (10 μg/ml). The BzATP-induced IL-1β release was significantly inhibited in the presence of the low molecular mass fraction collected after 10 min of stimulation with SLPI, and 30 min were needed for a full inhibition of the IL-1β release (Figure 11C).

### The Secretion of the Bioactive Factor Depends on Anx2 and iPLA2β

**Figure 12:**
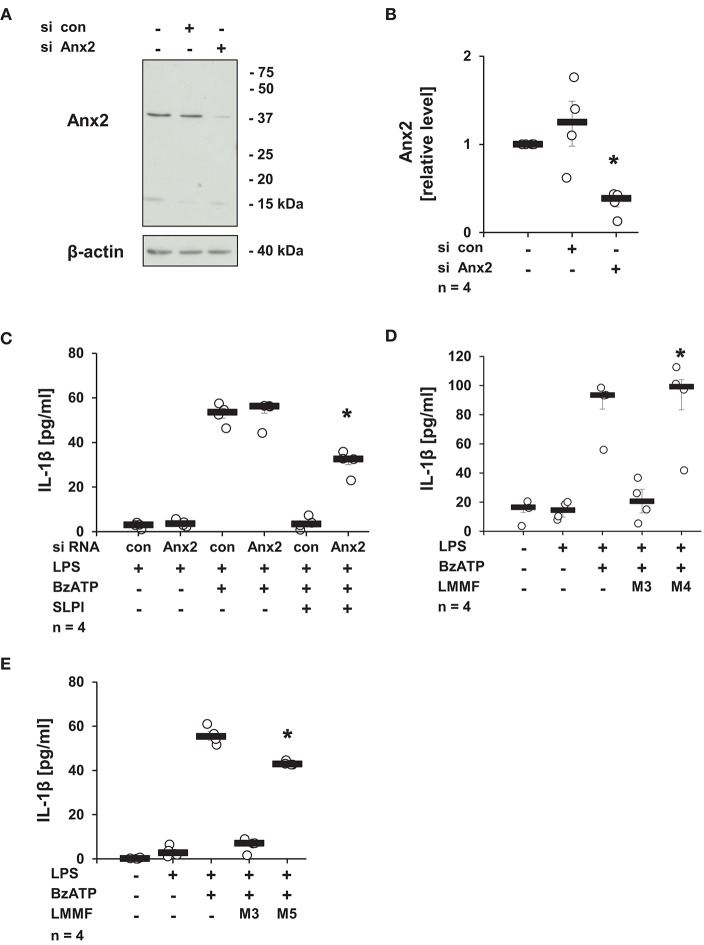
SLPI signaling involves annexin 2 (Anx2). Expression of Anx2 in U937 cells was diminished by siRNA. Cells were primed with LPS (1 μg/ml) for 5 h and further stimulated with 2′(3′)-O-(4-benzoylbenzoyl)adenosine 5′-triphosphate triethylammonium salt (BzATP; 100 mM) for 30 min in the presence or absence of SLPI (10 μg/ml). The efficiency and specificity of the knock-down was confirmed by immunoblotting. The concentration of IL-1β in the cell culture supernatant was measured by ELISA. **(A)** The protein expression level of Anx2 was silenced upon transfection with Anx2 siRNA; one representative immunoblot out of 4. **(B)** The optical density of the immuno-positive bands was measured and divided by the values obtained for β-actin on the same blot. The values gathered from untreated cells were set to one and all other values were calculated accordingly. ^*^p ≤ 0.05 significantly different compared to cells transfected with control (con) siRNA. **(C)** Down-regulation of the expression of Anx2 siRNA blunted the SLPI-induced inhibitory effect. ^*^p ≤ 0.05 significantly different compared to cells transfected with control siRNA (con) and stimulated with LPS, BzATP and SLPI. **(D,E)** Conditioned media were collected from U937 cells transfected with control siRNA (con) (M3), Anx2 siRNA (M4), or with iPLA2β siRNA (M5). All cells were primed before media collection with LPS for 5 h and SLPI was applied for additional 30 min. The low molecular mass fractions (LMMF) of M4 and M5 were devoid of inhibitory activity. ^*^p ≤ 0.05 significantly different compared to U937 cells treated with LMMF of M3. Experimental groups were compared by Kruskal-Wallis test followed by Mann-Whitney rank sum test.

Next, the involvement of Anx2, described as a membrane receptor/ binding protein for SLPI (52), in the production of the low molecular mass factor was determined. The expression of Anx2 in U937 cells was diminished using siRNA treatment, and the knock-down of the Anx2 protein was confirmed by immunoblotting followed by densitometry (Figures 12A,B). The knock-down of Anx2 did not affect the BzATP-mediated IL-1β secretion in the absence of SLPI, and the IL-1β release from U937 cells transfected with control non-target siRNA was fully abolished upon stimulation with SLPI, whereas the inhibition was significantly blunted in cells treated with siRNA targeting Anx2 (Figure 12C). To prove the involvement of Anx2 in the production of the inhibitory factor secreted in response to SLPI, the cells transfected with control siRNA or siRNA specific for Anx2 were primed with LPS for 5 h and SLPI was applied for 30 min. Conditioned media were collected and the inhibitory function of the low molecular mass fractions was tested. The low molecular mass fractions obtained from the cells transfected with control siRNA (M3) fully inhibited the BzATP-mediated IL-1β secretion, whereas fraction M4 collected from cells with reduced Anx2 expression had no effect on the IL-1β release (Figures 12D,E). In similar way, the relevance of iPLA2β for the production and release of the low molecular mass factor was tested. The low molecular weight fraction of conditioned media produced by cells transfected with siRNA specific for iPLA2β (M5) before stimulation with SLPI were devoid of any inhibitory activity (Figure 12E).

### The Inhibitory Function of the Low Molecular Mass Factor Is Blocked by nAChR Antagonists and by an Inhibitor of Src Kinase

**Figure 13:**
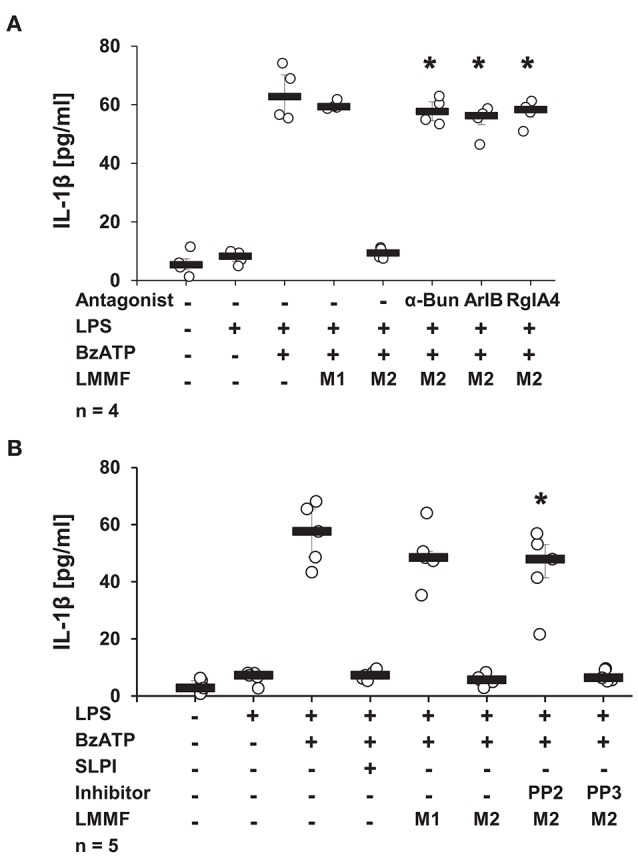
The bioactive factor signals via nicotinic acetylcholine receptor (nAChR) subunits α7, α9, and α10 and Src kinase. Human monocytic U937 cells were primed with LPS (1 μg/ml) for 5 h and cultured for additional 30 min in the absence (M1) or presence (M2) of SLPI (10 μg/ml). Conditioned media were collected and separated by ultrafiltration (cut-off 3 kDa) into high (HMMF) and low molecular mass fractions (LMMF). Conditioned medium M1 was supplemented with SLPI shortly before ultrafiltration. LPS-primed U937 cells were stimulated with 2′(3′)-O-(4-benzoylbenzoyl)adenosine 5′-triphosphate triethylammonium salt (BzATP; 100 mM) for 30 min in the presence or absence of M1 or M2. **(A)** Application of nicotinic antagonists α-bungarotoxin (α-Bun), RgIA4, or ArIB or **(B)** treatment with the Src kinase inhibitor PP2 abolished the inhibition mediated by the LMMF of M2. The inactive PP2 analogue PP3 had no effect on the inhibitory function of the LMMF of M2. ^*^p ≤ 0.05 significantly different compared to U937 cells treated with LPS, BzATP, and LMMF of M2. Experimental groups were compared by Kruskal-Wallis test followed by Mann-Whitney rank sum test.

Next, the involvement of nicotinic receptors in the inhibitory signaling mediated by the low molecular mass factor was tested. LPS-primed U937 cells were stimulated with SLPI (10 μg/ml) for 30 min, the cell-free supernatant was collected and ultrafiltrated to remove SLPI (M2). Low molecular mass fractions were applied to another set of LPS-primed U937 cells together with BzATP in the presence and absence of nicotinic antagonists. The release of IL-1β was measured 30 min later. As shown before, the low molecular weight fraction M2 inhibited the BzATP-induced IL-1β release. The inhibitory effect was antagonized in the presence of α-Bun, ArIB, and RgIA4 (Figure 13A). Similarly, the application of the Src kinase inhibitor PP2 fully abolished the inhibitory function of M2 fraction whereas the inactive control compound PP3 did not have any effect on BzATP-mediated IL-1β release (Figure 13B).

## Discussion

**Figure 14:**
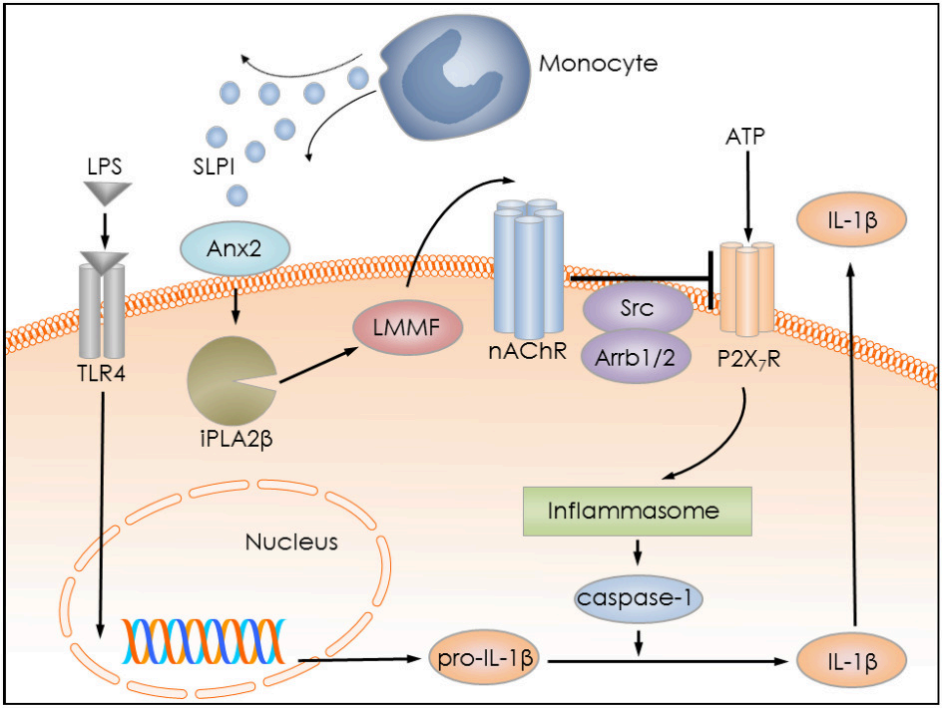
Graphical representation of the proposed inhibitory mechanism mediated by SLPI. LPS stimulates Toll-like receptor 4 (TLR4) at monocytic cells and induces the synthesis of pro-IL-1β. Extracellular ATP activates the P2X_7_R and leads to inflammasome assembly, caspase-1 activation and proteolytic cleavage of pro-IL-1β, and release of mature IL-1β. SLPI released for example from monocytes, activates iPLA2β in an annexin 2 (Anx2)-dependent manner and induces the production and release of an inhibitory low molecular mass factor (LMMF) that functions as an agonist of nicotinic acetylcholine receptors (nAChR). The LLMF in turn activates nACHRs containing subunits α7, α9, and α10, resulting in Src kinase activation, inhibition of P2X_7_R function, prevention of pro-IL-1β maturation, and IL-1β secretion.

Several pro-inflammatory stimuli, including LPS, IL-1β, and IL-6 have been identified to regulate SLPI expression by monocytes/macrophages (18–20). Anti-inflammatory properties of SLPI are well-documented by numerous publications (21, 53–55). They are mainly associated with a modulation of NF-κB signaling in monocytes/macrophages and a down-regulation of the gene expression of pro-inflammatory cytokines (29, 30, 56). We hypothesized that SLPI might rapidly control the release of monocytic IL-1β and protect against detrimental effects caused by elevated systemic levels of this cytokine. In the current study, we provided evidence for a novel anti-inflammatory mechanism mediated by SLPI, which results in a dose-dependent inhibition of the BzATP-induced IL-1β release by human and murine mononuclear leukocytes. The mRNA levels of pro-IL-1β are unimpaired in our experimental setting, probably due to the short incubation time with SLPI (30 min). SLPI in this experimental setting seems to signal via Anx2 and iPLA2β resulting in the quick release of small bioactive factors that, in turn, activate metabotropic functions of nAChRs containing subunits α7, α9 and α10, and lead to the inhibition of ATP-mediated currents and IL-1β release (Figure 14). This inhibitory mechanism is activated by both, the application of exogenous SLPI and by elevated endogenous SLPI expression.

Most experiments of the present study were performed on U937 cells, a human monocytic cell line that, like cell lines in general, does not fully reflect the properties of primary cells. To confirm the relevance of our findings, key experiments were repeated on PBMCs isolated from human or mouse blood. In line with our observations on U937 cells, stimulation of primary cells with BzATP resulted in the induction of IL-1β release independent on the source of the cells and in a reduced IL-1β release in the presence of SLPI. PBMCs from two out of eight human donors did not respond to SLPI for unknown reasons, whereas all mouse cells were SLPI-responsive. It might be that PBMCs from two non-responders requires optimization of SLPI concentration and this warrants further studies. Altogether, our results support the idea that the proposed mechanism is relevant *in vivo* and might play an important role in controlling the IL-1β release by blood mononuclear leukocytes.

In line with previously published data, LPS-primed U937 cells only released about 45 pg/ml IL-1β in response to stimulation with LPS and BzATP (10, 34, 35, 37). In contrast, the amount of IL-1β released from primary LPS-primed human PBMCs (around 2,000 pg/ml) was much higher compared to U937 cells. Mouse PBMCs were exclusively primed by the process of cell isolation and cultivation but not by LPS, and released IL-1β in about the same range like U937 cells. Hence, the absolute amount of IL-1β released in response to BzATP varies considerably depending on the cell type, *in vivo* and *in vitro* cell priming, and possibly depending on the species investigated.

To select the most effective inhibitory dose, different concentrations of SLPI were tested on U937 cells. Stimulation with 10 μg/ml of SLPI resulted in a full inhibition of the BzATP-mediated IL-1β release. This concentration of SLPI can be detected in saliva or in epithelial lining fluid under physiological conditions (21). The systemic concentrations of SLPI measured in blood serum of healthy volunteers are much lower, in average about 40 ng/ml, but can be induced under inflammatory conditions. For example, in the serum of septic surgical patients 132 ± 15 ng/ml of SLPI can be measured (22) and local concentrations near the inflammatory foci might be even higher.

We made considerable efforts to elucidate essential steps of the SLPI-mediated signaling cascade. In the view of the anti-protease activity of SLPI (23) and the ability of exogenous SLPI to cross the cell membrane (29), we initially hypothesized that SLPI might directly affect the function of the cytoplasmic protease caspase-1. To test this hypothesis, the IL-1β release by U937 cells was triggered by nigericin, a pore-forming bacterial toxin that activates caspase-1 in an ATP-independent manner (39). In contrast to this first hypothesis, the nigericin-induced IL-1β release was not inhibited by SLPI, suggesting that SLPI acts upstream of the NLRP3 inflammasome assembly in response to extracellular ATP.

Next, we investigated a potential direct inhibitory effect of SLPI on the P2X_7_R ion channel function using *Xenopus laevis* oocytes heterologously expressing the human P2X_7_R. However, SLPI did not impair BzATP-evoked ion currents, a result that argues against a direct SLPI-mediated modulation of P2X_7_R function. In U937 cells, however, SLPI inhibited ion current changes induced by extracellular ATP, most probably by an indirect inhibition of the monocytic P2X_7_R. In the following experiments were elucidated essential steps of the signaling cascade that eventually inhibits ATP signaling.

Previous reports from our group showed an involvement of iPLA2β in mechanisms controlling IL-1β release mediated by chemokines (38), β-nicotinamide adenine dinucleotide (10), and α1-antitrypsin (47). Therefore, we tested, whether iPLA2β plays an essential role in SLPI-mediated inhibition of IL-1β release. We observed that inhibitors of iPLA2β as well as silencing of the expression of iPLA2β significantly blunted the inhibitory function of SLPI. Similarly, mPBMCs obtained from *Pla2g6* gene-deficient mice were insensitive to SLPI. These results clearly confirmed a critical role of iPLA2β in the inhibitory mechanism mediated by SLPI.

It is known that iPLA2β activation leads to the release of free fatty acids, which can be further converted into diverse physiologically active lipid mediators (50). Furthermore, iPLA2β is involved in the generation of PC-containing metabolites with immunomodulatory properties (51). We postulated that the involvement of iPLA2β is associated with the production and the release of low molecular weight bioactive factors with inhibitory capacity. Indeed, conditioned medium of cells stimulated with SLPI inhibited the BzATP-mediated responses in a dose-dependent manner, although SLPI was depleted from the conditioned medium by ultrafiltration. In sharp contrast, conditioned medium from cells in which the expression of iPLA2β was silenced, was ineffective, suggesting that iPLA2β is required for the production or secretion of bioactive factors. Furthermore, collection of conditioned media at different time points after stimulation with SLPI clearly showed a time-dependent increase in the concentrations of the inhibitory factors, reaching the fully inhibitory concentration 30 min after SLPI application. This result seems to be in contrast to the observation that SLPI immediately blocks the IL-1β release. However, we assume that the inhibition is caused by autocrine factors and that the local concentrations surrounding individual cells rise quickly.

The inhibitory function of SLPI or conditioned medium was blocked by the nicotinic antagonists α-Bun, ArIB, and RgIA4, suggesting that activation of nAChRs containing subunits α7, α9/α10 is involved in signaling of SLPI. The role of these nAChR subunits in signaling of SLPI was further confirmed by gene-silencing and by using PBMCs from mice deficient in α7, α9, or α10 nAChR subunits. Recently, a novel cholinergic mechanism that inhibits the ATP-dependent release of IL-1β was discovered by our group (13–15). Several canonical and novel non-canonical cholinergic agonists were described to suppress the ATP-induced release of monocytic IL-1β via different ligand-specific combinations of nAChR subunits α7, α9, and α10 (13–15). Among them are lysophosphatidylcholine, the PLA2-dependent metabolite of phosphatidylcholine as well as glycerophosphocholine and PC. Signaling of SLPI seems to lead to the iPLA2β-dependent production of yet unidentified nAChR agonists that use the same nAChR combination (α7, α9, α10) as PC for signaling (14, 15). Nicotinic agonists activate metabotropic function of monocytic nAChRs without induction of canonical ionotropic receptor function in monocytes (13–15). SLPI did not induce changes in ion currents at nAChRs heterologously expressed in *Xenopus laevis* oocytes, whereas we showed that Src kinase and scaffolding proteins Arrbs are mandatory for the signaling down-stream of nAChRs. There are several reports that documented an involvement of nAChR antagonists and nAChRs in the activation of intracellular signaling cascades (44, 46, 57, 58). Interestingly, activation of nAChR α7 by nicotine induces cell proliferation by a mechanism involving Arrb-mediated activation of Src kinase (46). However, it is not clear, how Src kinase activation would modulate ATP-mediated responses. Unlike other P2X receptors, the P2X_7_R possesses a larger cytosolic C-terminus with 200 amino acid residues. The C-terminus of the P2X_7_R has been implicated in the regulation of receptor function, including signaling pathway activation, cellular localization, protein-protein interactions, and post-translational modification (59). Proteomic assays revealed a spectrum of cytosolic proteins interacting with the C-terminus of the P2X_7_R (60). Additionally, different kinases including PKC were reported to bind to the P2X_7_R (61), and proteins containing Src homology 3 (SH3) domains including Src kinase were predicted to interact with the C-terminus of P2X_7_R (62). Several putative phosphorylation sites were identified, and phosphorylation of some amino acids, for example Tyr 343, negatively regulate P2X_7_R function (60). Therefore, we postulate that Src kinase activated by cholinergic stimulation directly or indirectly leads to a posttranslational inhibitory modification of the P2X_7_R, to an inhibition of the ATP-mediated inflammasome assembly, and finally to an inhibition of IL-1β maturation and release.

The inhibitory function of SLPI was abolished upon down-regulation of Anx2 expression and conditioned medium produced in the absence of proper Anx2 expression was devoid of its inhibitory property. It is not clear how Anx2 contributes to the inhibitory responses mediated by SLPI. Anx2 is recognized as a pleiotropic, calcium-dependent and anionic phospholipid-binding protein that can exist as monomer and as a heterotetrameric complex with the plasminogen receptor protein, S100A10 (63, 64). Anx2 can be localized in the cytosol and on the cell surface (64). Cell surface Anx2, beside many other functions, has been suggested to act as a as a membrane receptor / binding protein for SLPI (52), and has been proposed to be involved in signal transduction (64). Based on our results, we postulate that SLPI binds to Anx2 on the cell surface and induces a yet undefined signaling cascade, which results in iPLA2β activation and in the release of the low molecular weight inhibitory factor (Figure 14).

Our study has several limitations. Although we discovered a novel anti-inflammatory function of SLPI, more research is needed to fully define the signaling pathway involved in the inhibitory mechanism. The major draw-back of this report is the lack of a clearly defined receptor for SLPI and the unknown identity of the low molecular weight inhibitory factors released in response to SLPI. In addition, the mechanism that links nAChR activation to the inhibition of the P2X_7_R function remains to be elucidated.

The recognition of the importance of IL-1β in the pathogenesis of different disorders already resulted in the development of diverse therapeutic strategies inhibiting the IL-1 system mainly for the treatment of diverse autoimmune diseases (2, 8). Unfortunately, targeting the IL-1 system in the context of trauma-induced SIRS and sepsis has not been beneficial up to now (2). This is probably due to the fact that host defense is impaired and infections are favored. Hence, anti-inflammatory treatment should selectively reduce circulating inflammasome-dependent mediators, without impairing local IL-1β release induced by invading pathogens. We postulate that SLPI, as a selective inhibitor of ATP-mediated responses, has the potential to be used in the clinic as a life-saving therapeutic agent for the treatment and prevention of trauma-induced SIRS and MODS, while sparing host defense against pathogens.

## Data Availability

All datasets generated for this study are included in the manuscript and/or the Supplementary Files.

## Author Contributions

AZ and VG were involved in research design, performance of experiments, interpretation of the data, and writing of the manuscript. DZ, KS, KR, JD, AA, and IM participated in research design, performance of experiments, interpretation of the data, and editing of the manuscript. AH was the investigating physician responsible for experiments on humans. GK-C, JMM, WC, RT, WP, and SJ contributed to research design, interpretation of the data, and editing of the manuscript.

### Conflict of Interest Statement

Certain conotoxins, including RgIA4 have been patented by the University of Utah; JM is an inventor on these patents. The remaining authors declare that the research was conducted in the absence of any commercial or financial relationships that could be construed as a potential conflict of interest.

## References

[1] Vladimer GI Marty-Roix R Ghosh S Weng D Lien E. Inflammasomes and host defenses against bacterial infections. Curr Opin Microbiol. (2013) 16:23–31. 10.1016/j.mib.2012.11.008 23318142 PMC3697846

[2] Dinarello CA Simon A van der Meer J W. Treating inflammation by blocking interleukin-1 in a broad spectrum of diseases. Nat Rev Drug Discov. (2012) 11:633–52. 10.1038/nrd3800 22850787 PMC3644509

[3] Guo H Callaway JB Ting JP. Inflammasomes: mechanism of action, role in disease, and therapeutics. Nat Med. (2015) 21:677–87. 10.1038/nm.3893 26121197 PMC4519035

[4] Dinarello CA Interleukin-1 in the pathogenesis and treatment of inflammatory diseases. Blood. (2011) 117:3720–32. 10.1182/blood-2010-07-273417 21304099 PMC3083294

[5] Cogswell JP Godlevski MM Wisely GB Clay WC Leesnitzer LM Ways JP Gray JG. NF-kappa B regulates IL-1 beta transcription through a consensus NF-kappa B binding site and a nonconsensus CRE-like site. J Immunol. (1994) 153:712–23. 8021507

[6] Gross O Thomas CJ Guarda G Tschopp J. The inflammasome: an integrated view. Immunol Rev. (2011) 243:136–51. 10.1111/j.1600-065X.2011.01046.x 21884173

[7] Rathinam VA Vanaja SK Fitzgerald KA. Regulation of inflammasome signaling. Nat Immunol. (2012) 13:333–42. 10.1038/ni.2237 22430786 PMC3523703

[8] Ozaki E Campbell M Doyle SL. Targeting the NLRP3 inflammasome in chronic inflammatory diseases: current perspectives. J Inflamm Res. (2015) 8:15–27. 10.2147/JIR.S51250 25653548 PMC4303395

[9] Cekic C Linden J. Purinergic regulation of the immune system. Nat Rev Immunol. (2016) 16:177–92. 10.1038/nri.2016.4 26922909

[10] Hiller SD Heldmann S Richter K Jurastow I Küllmar M Hecker A. Beta-nicotinamide adenine dinucleotide (beta-NAD) inhibits ATP-dependent IL-1beta release from human monocytic cells. Int J Mol Sci. (2018) 19:1126. 10.3390/ijms19041126 29642561 PMC5979475

[11] Savio LEB de Andrade Mello P da Silva CG Coutinho-Silva R The P2X7 receptor in inflammatory diseases: angel or demon? Front Pharmacol. (2018) 9:52 10.3389/fphar.2018.00052 29467654 PMC5808178

[12] Di Virgilio F Dal Ben D Sarti AC Giuliani AL Falzoni S The P2X7 receptor in infection and inflammation. Immunity. (2017) 47:15–31. 10.1016/j.immuni.2017.06.020 28723547

[13] Hecker A Küllmar M Wilker S Richter K Zakrzewicz A Atanasova S. Phosphocholine-modified macromolecules and canonical nicotinic agonists inhibit ATP-induced IL-1beta release. J Immunol. (2015) 195:2325–34. 10.4049/jimmunol.1400974 26202987

[14] Zakrzewicz A Richter K Agne A Wilker S Siebers K Fink B. Canonical and novel non-canonical cholinergic agonists inhibit ATP-induced release of monocytic interleukin-1beta via different combinations of nicotinic acetylcholine receptor subunits alpha7, alpha9, and alpha10. Front Cell Neurosci. (2017) 11:189. 10.3389/fncel.2017.00189 28725182 PMC5496965

[15] Richter K Mathes V Fronius M Althaus M Hecker A Krasteva-Christ G Phosphocholine - an agonist of metabotropic but not of ionotropic functions of alpha9-containing nicotinic acetylcholine receptors. Sci Rep. (2016) 6:28660 10.1038/srep28660 27349288 PMC4923896

[16] Richter K Sagawe S Hecker A Kullmar M Askevold I Damm J. C-reactive protein stimulates nicotinic acetylcholine receptors to control ATP-mediated monocytic inflammasome activation. Front Immunol. (2018) 9:1604. 10.3389/fimmu.2018.01604 30105015 PMC6077200

[17] Moreau T Baranger K Dade S Dallet-Choisy S Guyot N Zani ML. Multifaceted roles of human elafin and secretory leukocyte proteinase inhibitor (SLPI), two serine protease inhibitors of the chelonianin family. Biochimie. (2008) 90:284–95. 10.1016/j.biochi.2007.09.007 17964057

[18] Jin FY Nathan C Radzioch D Ding A. Secretory leukocyte protease inhibitor: a macrophage product induced by and antagonistic to bacterial lipopolysaccharide. Cell. (1997) 88:417–26. 9039268 10.1016/s0092-8674(00)81880-2

[19] Jin F Nathan CF Radzioch D Ding A. Lipopolysaccharide-related stimuli induce expression of the secretory leukocyte protease inhibitor, a macrophage-derived lipopolysaccharide inhibitor. Infect Immun. (1998) 66:2447–52. 9596701 10.1128/iai.66.6.2447-2452.1998 PMC108223

[20] Sallenave JM Shulmann J Crossley J Jordana M Gauldie J. Regulation of secretory leukocyte proteinase inhibitor (SLPI) and elastase-specific inhibitor (ESI/elafin) in human airway epithelial cells by cytokines and neutrophilic enzymes. Am J Respir Cell Mol Biol. (1994) 11:733–41. 10.1165/ajrcmb.11.6.7946401 7946401

[21] Weldon S McGarry N Taggart CC McElvaney NG. The role of secretory leucoprotease inhibitor in the resolution of inflammatory responses. Biochem Soc Trans. (2007) 35:273–6. 10.1042/BST0350273 17371258

[22] Grobmyer SR Barie PS Nathan CF Fuortes M Lin E Lowry SF. Secretory leukocyte protease inhibitor, an inhibitor of neutrophil activation, is elevated in serum in human sepsis and experimental endotoxemia. Crit Care Med. (2000) 28:1276–82. 10.1097/00003246-200005000-00003 10834665

[23] Williams SE Brown TI Roghanian A Sallenave JM. SLPI and elafin: one glove, many fingers. Clin Sci. (2006) 110:21–35. 10.1042/CS20050115 16336202

[24] Vogelmeier C Biedermann T Maier K Mazur G Behr J Krombach F. Comparative loss of activity of recombinant secretory leukoprotease inhibitor and alpha 1-protease inhibitor caused by different forms of oxidative stress. Eur Respir J. (1997) 10:2114–9. 9311513 10.1183/09031936.97.10092114

[25] Hiemstra PS. Defensins and cathelicidins in inflammatory lung disease: beyond antimicrobial activity. Biochem Soc Trans. (2006) 34:276–8. 10.1042/BST20060276 16545093

[26] Nukiwa T Suzuki T Fukuhara T Kikuchi T. Secretory leukocyte peptidase inhibitor and lung cancer. Cancer Sci. (2008) 99:849–55. 10.1111/j.1349-7006.2008.00772.x 18380788 PMC11159350

[27] Scott A Weldon S Taggart CC. SLPI and elafin: multifunctional antiproteases of the WFDC family. Biochem Soc Trans. (2011) 39:1437–40. 10.1042/BST0391437 21936829

[28] Ding A Thieblemont N Zhu J Jin F Zhang J Wright S. Secretory leukocyte protease inhibitor interferes with uptake of lipopolysaccharide by macrophages. Infect Immun. (1999) 67:4485–9. 10456890 10.1128/iai.67.9.4485-4489.1999 PMC96768

[29] Taggart CC Cryan SA Weldon S Gibbons A Greene CM Kelly E. Secretory leucoprotease inhibitor binds to NF-kappab binding sites in monocytes and inhibits p65 binding. J Exp Med. (2005) 202:1659–68. 10.1084/jem.20050768 16352738 PMC2212970

[30] Taggart CC Greene CM McElvaney NG O'Neill S Secretory leucoprotease inhibitor prevents lipopolysaccharide-induced ikappabalpha degradation without affecting phosphorylation or ubiquitination. J Biol Chem. (2002) 277:33648–53. 10.1074/jbc.M203710200 12084717

[31] Prompunt E Sanit J Barrere-Lemaire S Nargeot J Noordali H Madhani M. The cardioprotective effects of secretory leukocyte protease inhibitor against myocardial ischemia/reperfusion injury. Exp Ther Med. (2018) 15:5231–42. 10.3892/etm.2018.6097 29904407 PMC5996700

[32] Ashcroft GS Lei K Jin W Longenecker G Kulkarni AB Greenwell-Wild T. Secretory leukocyte protease inhibitor mediates non-redundant functions necessary for normal wound healing. Nat Med. (2000) 6:1147–53. 10.1038/80489 11017147

[33] Nakamura A Mori Y Hagiwara K Suzuki T Sakakibara T Kikuchi T. Increased susceptibility to LPS-induced endotoxin shock in secretory leukoprotease inhibitor (SLPI)-deficient mice. J Exp Med. (2003) 197:669–74. 10.1084/jem.20021824 12615907 PMC2193830

[34] Greene CM Carroll TP Smith SG Taggart CC Devaney J Griffin S. TLR-induced inflammation in cystic fibrosis and non-cystic fibrosis airway epithelial cells. J Immunol. (2005) 174:1638–46. 10.4049/jimmunol.174.3.1638 15661927

[35] Deng X Wang J Jiao L Utaipan T Tuma-Kellner S Schmitz G. iPLA2beta deficiency attenuates obesity and hepatic steatosis in ob/ob mice through hepatic fatty-acyl phospholipid remodeling. Biochim Biophys Acta. (2016) 1861:449–61. 10.1016/j.bbalip.2016.02.004 26873633

[36] Innocent N Livingstone PD Hone A Kimura A Young T Whiteaker P. Alpha-conotoxin arenatus IB[V11L,V16D] [corrected] is a potent and selective antagonist at rat and human native alpha7 nicotinic acetylcholine receptors. J Pharmacol Exp Ther. (2008) 327:529–37. 10.1124/jpet.108.142943 18664588 PMC2596936

[37] Romero HK Christensen SB Di Cesare Mannelli L Gajewiak J Ramachandra R Elmslie KS. Inhibition of alpha9alpha10 nicotinic acetylcholine receptors prevents chemotherapy-induced neuropathic pain. Proc Natl Acad Sci U.S.A. (2017) 114:E1825–832. 10.1073/pnas.1621433114 28223528 PMC5347537

[38] Amati AL Zakrzewicz A Siebers R Wilker S Heldmann S Zakrzewicz D. Chemokines (CCL3, CCL4, and CCL5) inhibit ATP-induced release of IL-1beta by monocytic cells. Mediators Inflamm. (2017) 2017:1434872. 10.1155/2017/1434872 28757683 PMC5516742

[39] Backhaus S Zakrzewicz A Richter K Damm J Wilker S Fuchs-Moll G. Surfactant inhibits ATP-induced release of interleukin-1beta via nicotinic acetylcholine receptors. J Lipid Res. (2017) 58:1055–66. 10.1194/jlr.M071506 28404637 PMC5454502

[40] Marchetti C Swartzwelter B Gamboni F Neff CP Richter K Azam T. OLT1177, a beta-sulfonyl nitrile compound, safe in humans, inhibits the NLRP3 inflammasome and reverses the metabolic cost of inflammation. Proc Natl Acad Sci U.S.A. (2018) 115:E1530–9. 10.1073/pnas.1716095115 29378952 PMC5816172

[41] Philip NS Carpenter LL Tyrka AR Price LH. The nicotinic acetylcholine receptor as a target for antidepressant drug development. ScientificWorldJournal. (2012) 2012:104105. 10.1100/2012/104105 22619570 PMC3349306

[42] McIntosh JM Absalom N Chebib M Elgoyhen AB Vincler M. Alpha9 nicotinic acetylcholine receptors and the treatment of pain. Biochem Pharmacol. (2009) 78:693–702. 10.1016/j.bcp.2009.05.020 19477168 PMC2739401

[43] Kudryavtsev D Shelukhina I Vulfius C Makarieva T Stonik V Zhmak M. Natural compounds interacting with nicotinic acetylcholine receptors: from low-molecular weight ones to peptides and proteins. Toxins. (2015) 7:1683–701. 10.3390/toxins7051683 26008231 PMC4448168

[44] King JR Gillevet TC Kabbani N. A G protein-coupled alpha7 nicotinic receptor regulates signaling and TNF-alpha release in microglia. FEBS Open Bio. (2017) 7:1350–61. 10.1002/2211-5463.12270 28904864 PMC5586346

[45] Kabbani N Nichols RA. Beyond the channel: metabotropic signaling by nicotinic receptors. Trends Pharmacol Sci. (2018) 39:354–66. 10.1016/j.tips.2018.01.002 29428175

[46] Dasgupta P Chellappan SP. Nicotine-mediated cell proliferation and angiogenesis: new twists to an old story. Cell Cycle. (2006) 5:2324–8. 10.4161/cc.5.20.3366 17102610

[47] Siebers K Fink B Zakrzewicz A Agne A Richter K Konzok S. Alpha-1 antitrypsin inhibits ATP-mediated release of interleukin-1beta via cd36 and nicotinic acetylcholine receptors. Front Immunol. (2018) 9:877. 10.3389/fimmu.2018.00877 29922281 PMC5996888

[48] Balsinde J Bianco ID Ackermann EJ Conde-Frieboes K Dennis EA. Inhibition of calcium-independent phospholipase A2 prevents arachidonic acid incorporation and phospholipid remodeling in P388D1 macrophages. Proc Natl Acad Sci U.S.A. (1995) 92:8527–31. 7667324 10.1073/pnas.92.18.8527 PMC41190

[49] Street IP Lin HK Laliberte F Ghomashchi F Wang Z Perrier H. Slow- and tight-binding inhibitors of the 85-kDa human phospholipase A2. Biochemistry. (1993) 32:5935–40. 8018213 10.1021/bi00074a003

[50] Burke JE Dennis EA. Phospholipase A2 biochemistry. Cardiovasc Drugs Ther. (2009) 23:49–59. 10.1007/s10557-008-6132-9 18931897 PMC2823292

[51] Ramanadham S Ali T Ashley JW Bone RN Hancock WD Lei X. Calcium-independent phospholipases A2 and their roles in biological processes and diseases. J Lipid Res. (2015) 56:1643–68. 10.1194/jlr.R058701 26023050 PMC4548770

[52] Ma G Greenwell-Wild T Lei K Jin W Swisher J Hardegen N. Secretory leukocyte protease inhibitor binds to annexin II, a cofactor for macrophage HIV-1 infection. J Exp Med. (2004) 200:1337–46. 10.1084/jem.20041115 15545357 PMC2211913

[53] Adapala VJ Buhman KK Ajuwon KM. Novel anti-inflammatory role of SLPI in adipose tissue and its regulation by high fat diet. J Inflamm. (2011) 8:5. 10.1186/1476-9255-8-5 21356117 PMC3051881

[54] Doumas S Kolokotronis A Stefanopoulos P. Anti-inflammatory and antimicrobial roles of secretory leukocyte protease inhibitor. Infect Immun. (2005) 73:1271–4. 10.1128/IAI.73.3.1271-1274.2005 15731023 PMC1064911

[55] Marino R Thuraisingam T Camateros P Kanagaratham C Xu YZ Henri J. Secretory leukocyte protease inhibitor plays an important role in the regulation of allergic asthma in mice. J Immunol. (2011) 186:4433–42. 10.4049/jimmunol.1001539 21335488 PMC3104396

[56] Greene CM McElvaney NG O'Neill SJ Taggart CC. Secretory leucoprotease inhibitor impairs toll-like receptor 2- and 4-mediated responses in monocytic cells. Infect Immun. (2004) 72:3684–7. 10.1128/IAI.72.6.3684-3687.2004 15155685 PMC415654

[57] Kabbani N Nordman JC Corgiat BA Veltri DP Shehu A Seymour VA. Are nicotinic acetylcholine receptors coupled to G proteins? Bioessays. (2013) 35:1025–34. 10.1002/bies.201300082 24185813

[58] King JR Nordman JC Bridges SP Lin MK Kabbani N. Identification and characterization of a G protein-binding cluster in alpha7 nicotinic acetylcholine receptors. J Biol Chem. (2015) 290:20060–70. 10.1074/jbc.M115.647040 26088141 PMC4536413

[59] Costa-Junior HM Sarmento Vieira F Coutinho-Silva RC. terminus of the P2X7 receptor: treasure hunting. Purinergic Signa. (2011) 7:7–19. 10.1007/s11302-011-9215-1 21484094 PMC3083127

[60] Kim M Jiang LH Wilson HL North RA Surprenant A. Proteomic and functional evidence for a P2X7 receptor signalling complex. EMBO J. (2001) 20:6347–58. 10.1093/emboj/20.22.6347 11707406 PMC125721

[61] Hung AC Chu YJ Lin YH Weng JY Chen HB Au YC. Roles of protein kinase c in regulation of P2X7 receptor-mediated calcium signalling of cultured type-2 astrocyte cell line, RBA-2. Cell Signal. (2005) 17:1384–96. 10.1016/j.cellsig.2005.02.009 15985361

[62] Denlinger LC Fisette PL Sommer JA Watters JJ Prabhu U Dubyak GR. Cutting edge: the nucleotide receptor P2X7 contains multiple protein- and lipid-interaction motifs including a potential binding site for bacterial lipopolysaccharide. J Immunol. (2001) 167:1871–6. 10.4049/jimmunol.167.4.1871 11489964

[63] Rescher U Gerke V. Annexins–unique membrane binding proteins with diverse functions. J Cell Sci. (2004) 117:2631–9. 10.1242/jcs.01245 15169834

[64] Bharadwaj A Bydoun M Holloway R Waisman D. Annexin A2 heterotetramer: structure and function. Int J Mol Sci. (2013) 14:6259–305. 10.3390/ijms14036259 23519104 PMC3634455

