# Supplementary material for: SLPI Inhibits ATP-Mediated Maturation of IL-1β in Human Monocytic Leukocytes: A Novel Function of an Old Player
Source: Front Immunol. 2019 Apr 4;10:664. doi: 10.3389/fimmu.2019.00664 (PMC6458293; doi:10.3389/fimmu.2019.00664)
Supplement: Supplementary file 1 [file Data_Sheet_1.PDF]

**Supplemental Table S1:** Cell death in U937 cells, human or mouse peripheral blood mononuclear cells (hPBMCs or mPBMCs) as measured by the lactate dehydrogenase (LDH) content in cell culture supernatants.

| | Cell treatment | Cell death [%] mean ±SD | n |
|---|---|---|---|
| Figure 1A | U937, - | 4.2 ± 2.3 | 4 |
| | U937, LPS | 4.2 ± 1.5 | 4 |
| | U937, LPS, BzATP | 2.9 ± 1.5 | 4 |
| | U937, LPS, BzATP, SLPI 0.01 µg/ml | 5.8 ± 3.5 | 4 |
| | U937, LPS, BzATP, SLPI 0.1 µg/ml | 5.8 ± 3.6 | 4 |
| | U937, LPS, BzATP, SLPI 1 µg/ml | 4.0 ± 1.9 | 4 |
| | U937, LPS, BzATP, SLPI 10 µg/ml | 4.1 ± 2.6 | 4 |
| Figure 1C | U937, - | 2.3 ± 0.6 | 4 |
| | U937, LPS | 1.4 ± 1.4 | 4 |
| | U937, LPS, BzATP | 2.0 ± 0.6 | 4 |
| | U937, LPS, BzATP, apyrase 0.5 U/ml | 1.7 ± 0.7 | 4 |
| Figure 1D | U937, - | 1.9 ± 0.5 | 4 |
| | U937, LPS | 3.0 ± 1.8 | 4 |
| | U937, LPS, nigericin 50 µM | 2.4 ± 0.9 | 4 |
| | U937, LPS, nigericin 50 µM, SLPI | 2.2 ± 0.9 | 4 |
| Figure 2A | hPBMCs, LPS | 2.1 ± 1.0 | 6 |
| | hPBMCs, LPS, BzATP | 2.9 ± 1.9 | 6 |
| | hPBMCs, LPS, BzATP, SLPI | 3.3 ± 1.2 | 6 |
| Figure 3B | U937, non-transfected, - | 4.5 ± 1.8 | 5 |
| | U937, non-transfected, LPS | 3.8 ± 0.9 | 5 |
| | U937, non-transfected, LPS, BzATP | 2.5 ± 0.9 | 5 |
| | U937, EV-transfected, - | 9.7 ± 3.0 | 5 |
| | U937, EV-transfected, LPS | 9.4 ± 3.4 | 5 |
| | U937, EV-transfected, LPS, BzATP | 8.9 ± 4.2 | 5 |
| | U937, SLPI-transfected, - | 8.5 ± 3.4 | 5 |
| | U937, SLPI-transfected, LPS | 9.7 ± 2.9 | 5 |
| | U937, SLPI-transfected, LPS, BzATP | 8.9 ± 3.0 | 5 |
| Figure 5A | U937, LPS | 1.2 ± 0.3 | 4 |
| | U937, LPS, BzATP | 1.1 ± 0.6 | 4 |
| | U937, LPS, BzATP, SLPI | 1.2 ± 0.4 | 4 |
| | U937, LPS, BzATP, SLPI, Mec 100 µM | 1.9 ± 0.7 | 4 |
| | U937, LPS, BzATP, SLPI, α-Bun 1 µM | 2.1 ± 1.2 | 4 |
| | U937, LPS, BzATP, SLPI 10, Stry 10 µM | 2.0 ± 1.5 | 4 |
| | LPS, BzATP, SLPI 10, ArIB 500 nM | 0.9 ± 0.3 | 4 |
| | LPS, BzATP, SLPI, RgIA4 200 nM | 1.1 ± 0.4 | 4 |

| | | | |
|---|---|---|---|
| Figure 5B | U937, con siRNA-transfected, LPS, BzATP | 2.7 ± 1.4 | 4 |
| | U937, α5 siRNA-transfected, LPS, BzATP | 2.3 ± 2.5 | 4 |
| | U937, α7 siRNA-transfected, LPS, BzATP | 4.5 ± 3.8 | 4 |
| | U937, α9 siRNA-transfected, LPS, BzATP | 5.3 ± 1.8 | 4 |
| | U937, α10 siRNA-transfected, LPS, BzATP | 11.9 ± 5.7 | 4 |
| | U937, con siRNA-transfected, LPS, BzATP, SLPI | 2.9 ± 1.3 | 4 |
| | U937, α5 siRNA-transfected, LPS, BzATP, SLPI | 4.2 ± 3.1 | 4 |
| | U937, α7 siRNA-transfected, LPS, BzATP, SLPI | 4.2 ± 3.9 | 4 |
| | U937, α9 siRNA-transfected, LPS, BzATP, SLPI | 4.8 ± 1.3 | 4 |
| | U937, α10 siRNA-transfected, LPS, BzATP, SLPI | 11.6 ± 5.8 | 4 |
| Figure 5C | mPBMCs WT, BzATP | 6.8 ± 2.5 | 5 |
| | mPBMCs $\alpha9^{-/-}$, BzATP | 8.0 ± 2.5 | 5 |
| | mPBMCs $\alpha10^{-/-}$, BzATP | 6.1 ± 2.5 | 5 |
| | mPBMCs WT, BzATP, SLPI | 6.1 ± 3.6 | 5 |
| | mPBMCs $\alpha9^{-/-}$, BzATP, SLPI | 8.0 ± 2.0 | 5 |
| | mPBMCs $\alpha10^{-/-}$, BzATP, SLPI | 6.2 ± 3.7 | 5 |
| Figure 7 | U937, LPS, BzATP | 1.7 ± 0.5 | 4 |
| | U937, LPS, BzATP, SLPI | 2.0 ± 1.3 | 4 |
| | U937, LPS, PP2 20 µM | 4.3 ± 2.2 | 4 |
| | U937, LPS, BzATP, PP2 20 µM | 3.6 ± 2.1 | 4 |
| | U937, LPS, BzATP, SLPI, PP2 1 µM | 2.3 ± 1.2 | 4 |
| | U937, LPS, BzATP, SLPI, PP2 5 µM | 2.0 ± 1.2 | 4 |
| | U937, LPS, BzATP, SLPI, PP2 10 µM | 2.8 ± 1.5 | 4 |
| | U937, LPS, BzATP, SLPI, PP2 20 µM | 3.6 ± 1.3 | 4 |
| | U937, LPS, BzATP, PP3 20 µM | 1.5 ± 0.7 | 4 |
| | U937, LPS, BzATP, SLPI, PP3 20 µM | 2.4 ± 0.8 | 4 |
| Figure 8E | U937, con siRNA-transfected, LPS, BzATP | 4.5 ± 4.6 | 4 |
| | U937, Arrb1 siRNA-transfected, LPS, BzATP | 6.0 ± 6.0 | 3 |
| | U937, Arrb2 siRNA-transfected, LPS, BzATP | 5.5 ± 5.7 | 3 |
| | U937, Arrb1/2 siRNA-transfected, LPS, BzATP | 8.5 ± 7.1 | 3 |
| | U937, con siRNA-transfected, LPS, BzATP, SLPI | 5.2 ± 5.8 | 4 |
| | U937, Arrb1 siRNA-transfected, LPS, BzATP, SLPI | 5.8 ± 5.3 | 3 |
| | U937, Arrb2 siRNA-transfected, LPS, BzATP, SLPI | 5.0 ± 5.0 | 3 |
| | U937, Arrb1/2 siRNA-transfected, LPS, BzATP, SLPI | 8.9 ± 7.5 | 3 |
| Figure 9A | U937, LPS | 2.5 ± 1.6 | 4 |
| | U937, LPS, BzATP | 1.6 ± 0.8 | 4 |
| | U937, LPS, BzATP, SLPI | 1.6 ± 0.9 | 4 |
| | U937, LPS, BzATP, ATK 50 µM | 2.4 ± 0.5 | 4 |
| | U937, LPS, BzATP, SLPI, ATK 50 µM | 2.5 ± 0.8 | 4 |
| Figure 9B | U937, LPS | 2.3 ± 0.7 | 4 |
| | U937, LPS, BzATP | 2.3 ± 0.7 | 4 |
| | U937, LPS, BzATP, SLPI | 2.4 ± 0.7 | 4 |
| | U937, LPS, BzATP, BEL 50 µM | 1.6 ± 0.2 | 4 |
| | U937, LPS, BzATP, SLPI, BEL 50 µM | 1.0 ± 0.5 | 4 |

| | | | |
|---|---|---|---|
| Figure 9D | U937, con siRNA-transfected, LPS | 3.6 ± 1.9 | 4 |
| | U937, iPLA2β siRNA transfected, LPS | 3.2 ± 2.7 | 4 |
| | U937, con siRNA-transfected, LPS, BzATP | 2.9 ± 1.4 | 4 |
| | U937, iPLA2β siRNA-transfected, LPS, BzATP | 3.6 ± 2.3 | 4 |
| | U937, con siRNA-transfected, LPS, BzATP, SLPI | 2.9 ± 2.4 | 4 |
| | U937, iPLA2β siRNA-transfected, LPS, BzATP, SLPI | 3.6 ± 1.5 | 4 |
| Figure 9E | mPBMCs WT, BzATP | 3.4 ± 1.0 | 4 |
| | mPBMCs *pla2g6*$^{-/-}$, BzATP | 10.8 ± 4.7 | 4 |
| | mPBMCs WT, BzATP, SLPI | 2.4 ± 0.9 | 4 |
| | mPBMCs *pla2g6*$^{-/-}$, BzATP, SLPI | 10.0 ± 3.8 | 4 |
| Figure 10B | U937, - | 3.0 ± 1.0 | 4 |
| | U937, LPS | 2.9 ± 0.8 | 4 |
| | U937, LPS, BzATP | 3.0 ± 0.5 | 4 |
| | U937, LPS, BzATP, SLPI | 3.0 ± 1.0 | 4 |
| | U937, LPS, BzATP, SLPI, LMMF M1 100% | 3.9 ± 0.8 | 4 |
| | U937, LPS, BzATP, SLPI, LMMF M2 6.25% | 3.4 ± 0.9 | 4 |
| | U937, LPS, BzATP, SLPI, LMMF M2 12.5% | 3.4 ± 0.5 | 4 |
| | U937, LPS, BzATP, SLPI, LMMF M2 25% | 4.0 ± 0.2 | 4 |
| | U937, LPS, BzATP, SLPI, LMMF M2 50% | 3.7 ± 0.6 | 4 |
| | U937, LPS, BzATP, SLPI, LMMF M2 100% | 4.3 ± 0.4 | 4 |
| Figure 10C | U937, - | 2.8 ± 0.4 | 4 |
| | U937, LPS | 3.4 ± 0.5 | 4 |
| | U937, LPS, BzATP | 2.6 ± 0.2 | 4 |
| | U937, LPS, BzATP, SLPI | 3.0 ± 0.1 | 4 |
| | U937, LPS, BzATP, SLPI, LMMF M2 5 min | 2.4 ± 0.6 | 4 |
| | U937, LPS, BzATP, SLPI, LMMF M2 10 min | 2.6 ± 0.8 | 4 |
| | U937, LPS, BzATP, SLPI, LMMF M2 15 min | 2.9 ± 1.5 | 4 |
| | U937, LPS, BzATP, SLPI, LMMF M2 20 min | 3.6 ± 0.7 | 4 |
| | U937, LPS, BzATP, SLPI, LMMF M2 30 min | 2.8 ± 0.9 | 4 |
| Figure 11C | U937, con siRNA-transfected, LPS | 5.8 ± 2.5 | 4 |
| | U937, Anx2 siRNA-transfected, LPS | 4.4 ± 1.1 | 4 |
| | U937, con siRNA-transfected, LPS, BzATP | 5.5 ± 2.1 | 4 |
| | U937, Anx2 siRNA-transfected, LPS, BzATP | 3.8 ± 1.0 | 4 |
| | U937, con siRNA-transfected, LPS, BzATP, SLPI | 5.3 ± 2.4 | 4 |
| | U937, Anx2 siRNA-transfected LPS, BzATP, SLPI | 4.3 ± 1.1 | 4 |
| Figure 11D | U937, - | 1.6 ± 0.3 | 4 |
| | U937, LPS | 1.9 ± 0.4 | 4 |
| | U937, LPS, BzATP | 1.7 ± 0.5 | 4 |
| | U937, LPS, BzATP, SLPI, LMMF M3 | 2.2 ± 0.4 | 4 |
| | U937, LPS, BzATP, SLPI, LMMF M4 | 2.2 ± 0.4 | 4 |

| | | | |
|---|---|---|---|
| Figure 11E | U937, - | 2.0 ± 0.5 | 4 |
| | U937, LPS | 2.0 ± 0.6 | 4 |
| | U937, LPS, BzATP | 1.9 ± 0.4 | 4 |
| | U937, LPS, BzATP, SLPI, LMMF M3 | 2.4 ± 0.9 | 4 |
| | U937, LPS, BzATP, SLPI, LMMF M5 | 2.2 ± 0.5 | 4 |
| Figure 12A | U937, - | 2.9 ± 0.8 | 4 |
| | U937, LPS | 3.6 ± 1.1 | 4 |
| | U937, LPS, BzATP | 3.0 ± 1.3 | 4 |
| | U937, LPS, BzATP, LMMF M1 | 2.5 ± 1.2 | 4 |
| | U937, LPS, BzATP, LMMF M2 | 2.7 ± 1.5 | 4 |
| | U937, LPS, BzATP, LMMF M2, α-Bun 1 µM | 2.7 ± 1.6 | 4 |
| | U937, LPS, BzATP, LMMF M2, ArIB 500 µM | 2.7 ± 1.5 | 4 |
| | U937, LPS, BzATP, LMMF M2, RgIA4 200 µM | 3.2 ± 1.1 | 4 |
| Figure 12B | U937, - | 3.3 ± 1.5 | 5 |
| | U937, LPS | 3.2 ± 1.1 | 5 |
| | U937, LPS, BzATP | 2.9 ± 0.8 | 5 |
| | U937, LPS, BzATP, SLPI | 3.5 ± 1.2 | 5 |
| | U937, LPS, BzATP, LMMF M1 | 4.0 ± 3.2 | 5 |
| | U937, LPS, BzATP, LMMF M2 | 2.2 ± 0.3 | 5 |
| | U937, LPS, BzATP, LMMF M2, PP2 20 µM | 2.9 ± 0.5 | 5 |
| | U937, LPS, BzATP, LMMF M2, PP3 20 µM | 2.7 ± 0.3 | 5 |

Cell death was estimated via measurement of the release of lactate dehydrogenase (LDH) into the cell culture medium. The data depicted in this table correspond to the experiments shown in the respective figures of the main part of this manuscript. Human monocytic U937 cells were primed with lipopolysaccharide (LPS, 1 µg/ml, for 5 h) and further stimulated with 2´(3´)-O-(4-benzoylbenzoyl)adenosine 5´-triphosphate triethylammonium salt (BzATP; 100 mM); hPBMCs were pulsed with LPS (5 ng/ml). Secretory leukocyte protease inhibitor (SLPI) was applied at a concentration of 10 µg/ml unless stated differently. The concentration of diverse inhibitors or antagonists is indicated in the table. ARRB1, arrestin β1; ARRB2, arrestin β2; Anx2, annexin 2; ATK, arachidonyl trifluoromethyl ketone; α-Bun, α-bungarotoxin; α5, CHRNA5; α9, CHRNA 9; α10, CHRNA10; BEL, bromoenol lactone; EV, empty vector; iPLA2β, calcium-independent phospholipase A2β; LMMF, low molecular mass fraction, M1, conditioned medium collected from U937 cells upon stimulation with LPS; M2, conditioned medium collected from U937 cells upon stimulation with LPS and SLPI; M3, conditioned medium collected from U937 cells transfected with control siRNA and stimulated with LPS and SLPI; M4, conditioned medium collected from U937 transfected with Anx2 siRNA and stimulated with LPS and SLPI; M5, conditioned medium collected from U937 cells transfected with iPLA2β siRNA and stimulated with LPS and SLPI; Mec, mecamylamine hydrochloride; PP2, 4-amino-5-(4-chlorophenyl)-7-(t-butyl)pyrazolo[3,4-d]pyrimidine; PP3, 4-amino-7-phenylpyrazolo[3,4-d]pyrimidine; Stry, strychnine; WT, wild-type.
